# Multi-objective design and optimization of squeezed branch pile based on orthogonal test

**DOI:** 10.1038/s41598-023-49936-y

**Published:** 2023-12-15

**Authors:** Ziqi Wang, Cunbao Zhao, Wenyue Zhang

**Affiliations:** 1 State Key Laboratory of Mechanical Behavior and System Safety of Traffic Engineering Structures, Shijiazhuang Tiedao University, Shijiazhuang, 050043 China; 2 Hebei Engineering Innovation Center for Traffic Emergency and Guarantee, Shijiazhuang Tiedao University, Shijiazhuang, 050043 China; 3 School of Safety Engineering and Emergency Management, Shijiazhuang Tiedao University, Shijiazhuang, 050043 China

**Keywords:** Engineering, Civil engineering

## Abstract

In recent years, the emergence of the squeezed branch pile has presented a new avenue for civil engineering, offering a distinctive structure and favorable mechanical characteristics. Squeezed branch piles have strong compressive, uplift, and horizontal load resistance capabilities. Due to the existence of discs, the geometric parameters of squeezed branch piles are abundant but important. This article selects number of discs, disc diameter, disc squeeze angle, and disc spacing as the main influencing factors on the bearing capacity of squeezed branch piles and conducts a qualitative analysis of their mechanical properties. The aim of this article is to analyze the different bearing performances of squeezed branch piles through orthogonal experimental design, simulate test conditions using finite element software ABAQUS, obtain relevant data, and finally determine the weight ranking and optimal combination of influencing factors through range analysis to provide better guidance for engineering practices. Through multi-objective optimization design, six optimization objectives including compressive performance, compressive economic efficiency, uplift performance, uplift economic efficiency, maximum horizontal displacement and maximum bending moment of pile body were analyzed. The analysis methods used included comprehensive balance method, queue scoring method, principal component analysis method, entropy weight method, and analytic hierarchy process. The conclusions obtained are similar, and based on the judgment, the squeezed branch pile with 4 discs, disc diameter of 2.5D, disc squeeze angle of 35°, and disc spacing of 3D is considered as the optimal combination under consideration of all optimization objectives.

## Introduction

Nowadays, with the accelerated urbanization and the increasing human activities such as buildings and transportation facilities, land resources have become more and more scarce. Meanwhile, due to natural disasters and geological disasters, the soil conditions in many areas are not ideal, such as soft soil layers and karst strata. This requires us to adopt more efficient foundation treatment technology in the construction process to meet the requirements of buildings and transportation facilities in terms of foundation bearing capacity, seismic capacity and liquefaction resistance. In order to make pile foundations provide higher bearing capacity, pile lateral friction resistance and pile bottom end bearing capacity are the two main areas of research conducted by scholars. In recent years, a large number of scholars have studied various aspects of different pile foundations in different soil conditions^1–5^, of which experimental studies and numerical tests are the main ones, and Ateş, B., Şadoğlu, E. also studied group efficiency with pile groups as the main research object^6^.

Traditional foundation treatment methods (such as cast-in-place piles, steel pipe piles, etc.) have problems such as restricted application, difficult construction and high cost. For example, cast-in-place piles are only applicable to soft soil layer and gravel soil layer, while it is very difficult to construct in rock layer; the recovery and disposal of steel pipe piles are troublesome, and their construction process will produce noise and vibration and other problems that affect the surrounding environment and residents' life. These problems provide opportunities for the research of new foundation treatment technology.

In this context, the research and application of new pile foundations such as squeezed branch piles^7^ and bamboo piles^8^ came into being. the earliest prototype of squeezed branch piles was proposed and tested in the 1950s. In the 1960s and 1970s, India, Britain and the Soviet Union took the lead in using multi-sectional expanded piles^9^ in different soils such as black cotton, loess and sub-clay, and carried out many model tests and field tests. In the late 1980s, China started to research on the squeezed branch piles, and Beijing Junhua Foundation Engineering Group applied for the patent of squeezed branch pile technology in the 1990s^10^.

It is generally believed that the vertical bearing capacity of squeezed branch pile is composed of the pile side frictional resistance, pile bottom end resistance and disc end resistance, and the bearing capacity can be calculated according to the specific parameters and empirical coefficients of the soil and disc^11^. Yuwen Ju^12^ observed and recorded the pile lateral frictional resistance, pile bottom end resistance and disc end resistance by single pile static load test, and improved the theory of compressive load bearing of squeezed branch pile. By compiling the data, it is found that the pile side frictional resistance has reached its limit at the late stage of static load loading and no longer increases with the increase of load, and more than 50% of the external load is borne by the supporting disc; the resistance provided by the supporting disc of the squeezed branch pile has the nature of end-bearing force, so the squeezed branch pile can be characterized as frictional end-bearing pile.

Xiaojuan Gao^13^ used ABAQUS to analyze the bearing capacity of squeezed branch piles as affected by different soil parameters, including the internal friction angle of the soil around the pile, cohesion, pile-soil friction coefficient and elastic modulus of different soils around the pile. Chen Fei^14^ found in an engineering example that there is a significant time series effect on the play of pile lateral frictional resistance and disc resistance, and the squeezed branch pile has 89% higher bearing capacity and 28% less cost than the equal section pile of the same diameter and length. Yili Wang^15^ analyzed the bearing capacity, settlement and axial force distribution of the squeezed branch pile by finite element simulation, and found that the pile axial force produced abrupt changes at the upper and lower interfaces of the disc, and obtained the optimal disc spacing, which was about 2.5 to 3 times the diameter of the disc. The displacement and stress fields of the squeezed branch pile were analyzed by Yabin Xi, and it was found that under the load, a "tension crack zone"^16^ would be formed between the pile and the upper soil, resulting in no actual contact between the two.

In this paper, we calculate the weights of various indicators of the squeezed branch pile based on the comprehensive balance method, queuing scoring method, principal component analysis, entropy weight method and analytic hierarchy process, and analyze the optimal test combination under the comprehensive consideration of all indicators.

## Orthogonal experiment of squeezed branch piles

**Figure 1:**
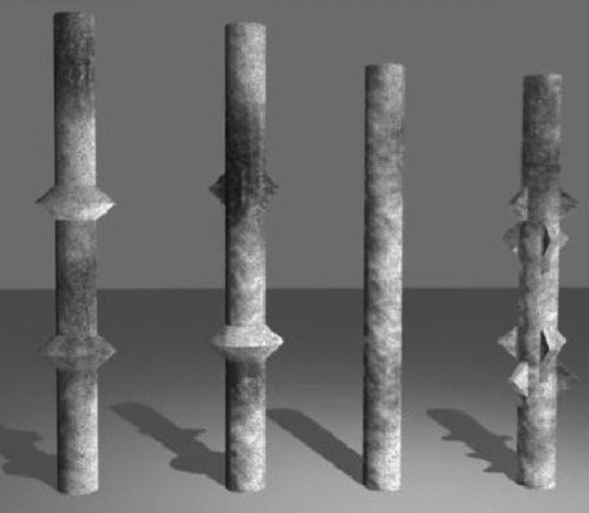
Structure of squeezed branch piles.

The squeezed branch pile has a special disc or branch structure^17^, as shown in Fig. 1, which requires a disc formation process during pile formation to ensure its load-bearing performance and stability. As a kind of special-shaped cross-section pile, squeezed branch pile has more geometric parameters to be considered in its design than ordinary straight piles. Combined with the application of squeezed branch piles in engineering practice, it is further found that there are many parameters that can be adjusted during the design of squeezed branch piles^18–20^, such as diameter of pile, length of pile, position of disc, spacing of disc, squeeze angle of disc, number of disc, diameter of disc, etc.

These parameters are very important, and their changes will inevitably affect the bearing capacity of the pile. In order to explore how these factors affect the bearing performance of the pile foundation, several factors that are most closely related to the disc structure are selected for orthogonal experimental design.

Orthogonal experimental design is a multi-factor and multi-level experimental design method, which aims to determine the optimal parameter combination through a limited number of experiments to achieve the optimization goal. It is an efficient, fast and economical experimental design method, which can greatly reduce the cost and time of experiments^21–23^.

According to engineering practice and theoretical needs, number of discs, disc diameter, disc squeeze angle and disc spacing are selected as the factors of this orthogonal experiment^24–26^. The specific levels of the four factors are shown in the Table 1. Among them, the level values corresponding to each factor can be determined according to the actual needs. In this paper, the diameter and pile length of squeezed branch piles are set for the purpose of making model piles, so the diameter (D) of the model squeezed branch pile is 30mm, and the pile length is 600mm.

**Table 1:** Level table of orthogonal experiment factors (D is the diameter of the pile).

Levels	Factors			
	Number of discs	Disc diameter	Disc squeeze angle	Disc spacing
1	2	1.5D	35°	2D
2	3	2D	40°	2.5D
3	4	2.5D	45°	3D

Construct the orthogonal test table according to specific factors and levels, and generate Table 2 Orthogonal test table.

**Table 2:** Orthogonal test table L9(3^4^).

Test numbers	Factors			
	Number of discs	Disc diameter	Disc squeeze angle	Disc spacing
1	2	1.5D	35°	2D
2	2	2D	40°	2.5D
3	2	2.5D	45°	3D
4	3	1.5D	40°	3D
5	3	2D	45°	2D
6	3	2.5D	35°	2.5D
7	4	1.5D	45°	2.5D
8	4	2D	35°	3D
9	4	2.5D	40°	2D

**Figure 2:**
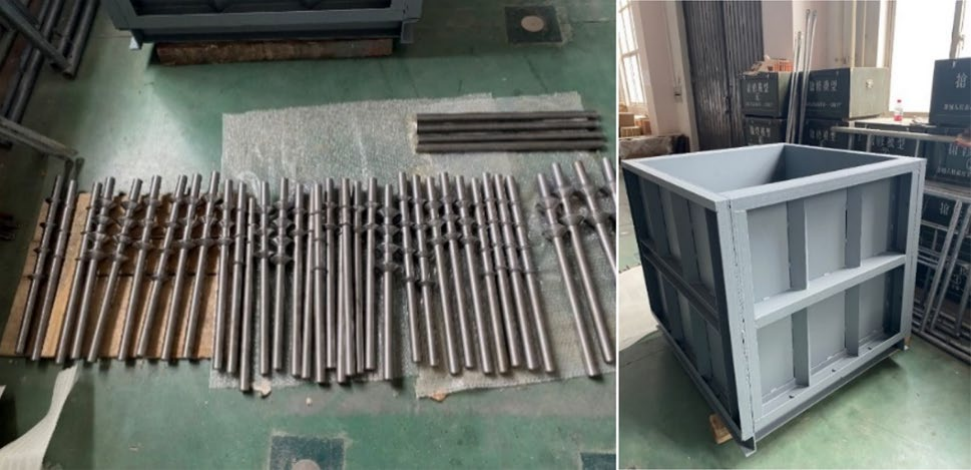
Model pile and box.

In the test, the effective pile length was set to 500mm, and the top of the pile protruded 100 mm from the surface of the soil layer to facilitate the application of load. Considering the scale of the model test and the impact of boundary effects, the model box is designed as a cube with a side length of 1 m, the horizontal boundary exceeds 20 times the diameter of the supporting plate model pile, and the vertical boundary is 1 times the effective pile length. The bottom plate and surrounding steel plates are both 8 mm thick, and each steel plate is welded into a whole. The bottom plate and surrounding steel plates of the model box are reinforced by channel steel, as shown in Fig. 2.

The soil used for the test comes from the construction site of the Science and Technology Building of Shijiazhuang Tiedao University. After the undisturbed soil is retrieved, some soil samples are taken for drying, and the soil is analyzed according to the soil test standards to obtain the soil parameters, as shown in Table 3.

**Table 3:** Pile and soil parameters.

	Density/g cm^(−3)	Specific gravity of solid particles	Moisture content/%	Cohesion/kPa	Internal friction angle/°	Modulus of elasticity/MPa	Poisson's ratio
Soil	2.185	2.75	14.70	3.1	29.362	2.353	0.3
Pile	7.85	–	–	–	–	210,000	0.3

## Finite element analysis of compressive resistance capabilities of squeezed branch piles

In Chapter 2, nine different geometrical parameters of squeezed branch piles are obtained by conducting orthogonal experimental designs. In order to further investigate and verify the conclusions about the regularity of the compressive performance of the squeezed branch piles, this chapter will first perform numerical simulations of the compressive performance of the nine types of piles by using the finite element software ABAQUS^27,28^ for the analysis. The results of the orthogonal test design are analyzed, and two software programs, IBM SPSS Statistics and Orthogonal Design Assistant, are used for data processing and analysis, and then the optimal or better combination of factor levels is determined, and the weights of different factors on the test results are ranked to summarize the major and minor factors affecting the vertical compressive performance of piles.

### Finite element modeling

#### Geometric modeling

**Figure 3:**
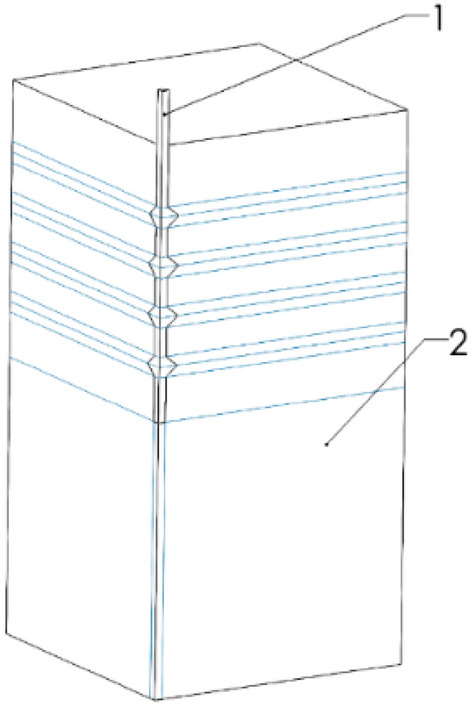
1/4 model(1:pile, 2:soil).

In order to simulate the working behavior of the squeezed branch pile under the action of vertical load, a three-dimensional solid model is used for simulation in this paper. When establishing the geometric model, according to the force of the model, use the principle of symmetry to take 1/4 model for simulation, as shown in Fig. 3.

What needs to be additionally explained is that this paper focuses on the influence of various parameters of the squeezed branch piles on the bearing capacity of the pile, and does not consider the process of pile penetration into the soil body or the unevenness of the soil body. The soil around the pile is in ideal condition and the default condition of the soil for all the working conditions used is consistent. The final point of this paper is to provide some reference to the actual project by observing the different performance of different piles in the same environment.

#### Pile and soil principal structure model

In the pile-soil action, the squeezed branch pile is subjected to external load and the soil around the pile is damaged, while the pile itself is stiff and almost not damaged under the external load, and this paper is not about the damage analysis of the squeezed branch pile, so this paper simulates the use of the linear elastic model for the squeezed branch pile.

The tensor type intrinsic relationship of the linear elastomer is

1 $$ \sigma_{ij} = \left[ {\frac{2G\mu }{{1 - 2\mu }}\delta_{ij} \delta_{kl} + G\left( {\delta_{ik} \delta_{jl} + \delta_{il} \delta_{jk} } \right)} \right]\varepsilon_{kl} = D_{ijkl} \varepsilon_{kl} $$

where $D_{ijkl}$ is the elastic tensor component, $\mu$ is the Poisson's ratio of the material, and $E$ is the modulus of elasticity of the material. The matrix type of $D_{ijkl}$ is shown in Eq. (2), and the formula of G is shown in Eq. (3).

2 $$ [D] = \frac{E}{(1 + \mu )(1 - 2\mu )}\left[ {\begin{array}{*{20}c} {1 - \mu } & \mu & \mu & 0 & 0 & 0 \\ {} & {1 - \mu } & \mu & 0 & 0 & 0 \\ {} & {} & {1 - \mu } & 0 & 0 & 0 \\ {} & {} & {} & {\frac{1}{2} - \mu } & 0 & 0 \\ {} & {{\text{sym}}} & {} & {} & {\frac{1}{2} - \mu } & 0 \\ {} & {} & {} & {} & {} & {\frac{1}{2} - \mu } \\ \end{array} } \right] $$

3 $$ G = \frac{E}{2(1 + \mu )} $$

This simulation uses the Mohr–Coulomb model for soils, which is used to describe the strength and damage behavior of soils and is widely used in geotechnical engineering. The model is based on the elasto-plastic theory and uses a simple linear relationship to describe the shear strength of the soil.

The expression of the three-dimensional stress space for the Mohr–Coulomb yielding condition is given by:

4 $$ \frac{1}{3}I_{1} \sin \varphi + \sqrt {J_{2} } \sin \left( {\theta + \frac{\pi }{3}} \right) + \frac{{\sqrt {J_{2} } }}{\sqrt 3 }\cos \left( {\theta + \frac{\pi }{3}} \right)\sin \varphi - C\cos \varphi = 0 $$

where $\theta$ is determined by $\cos 3\theta = \sqrt {2J_{3} /\tau_{8}^{3} }$, $I_{1}$ is the first invariant of the stress tensor, $J_{2}$ and $J_{3}$ are the second and third invariants of the stress deflection tensor, and $\tau_{8}^{{}}$ is the octahedral shear stress.

**Figure 4:**
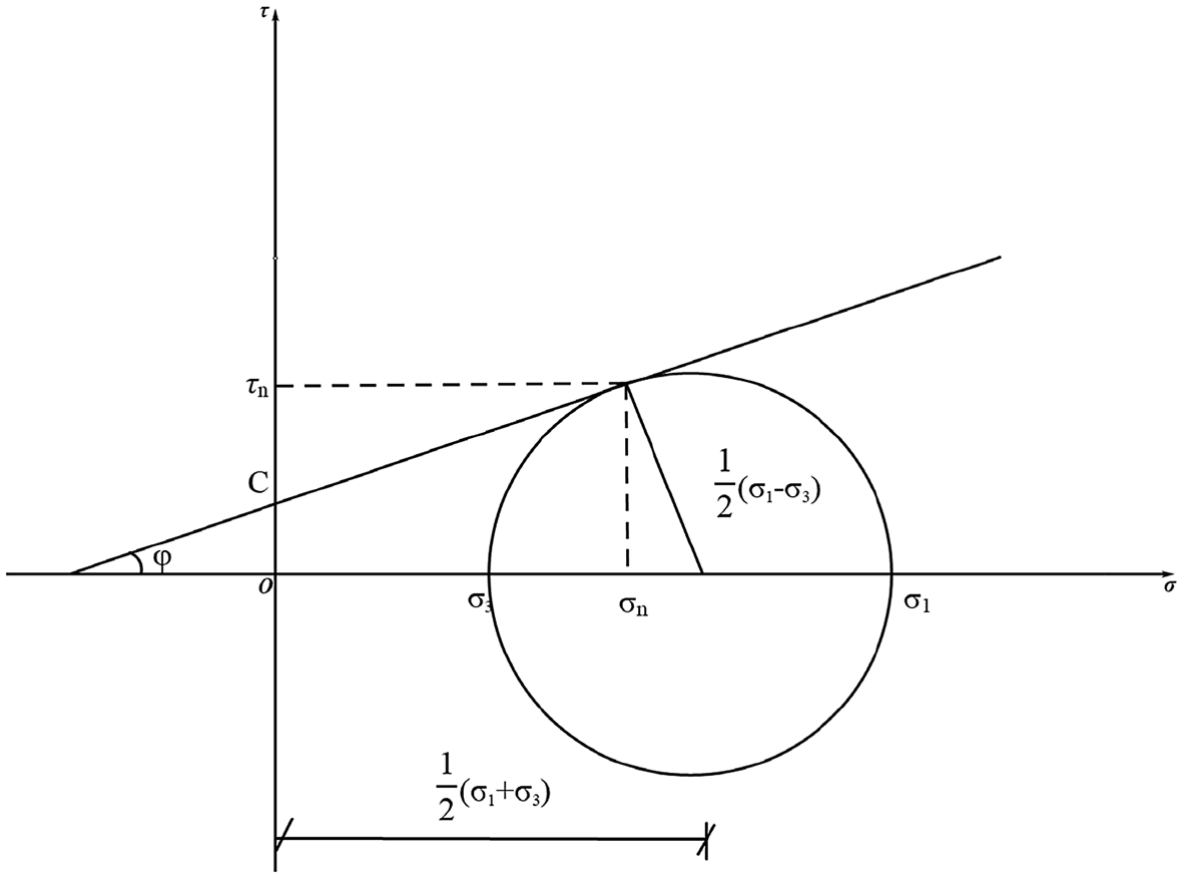
Mohr stress circle.

The ultimate shear strength of a soil on any of its bearing surfaces can be expressed by the Mohr–Coulomb law, as shown in Fig. 4, and calculated as:

5 $$ \tau_{{\text{n}}} = c + \sigma_{n} \tan \varphi $$

where $c$ is the cohesive force, numerically equal to the intercept of the damage line on the vertical axis, $\sigma_{n}$ is the positive stress on the force surface, and $\varphi$ is the angle of internal friction of the soil.

According to the Mohr stress circle Fig. 4 of the soil, it is obtained that:

6 $$ \tau_{{\text{n}}} = R\cos \varphi $$

7 $$ \sigma_{n} = \frac{1}{2}\left( {\sigma_{x} + \sigma_{y} } \right) - R\sin \varphi $$

8 $$ R = c\cos \varphi - \frac{1}{2}\left( {\sigma_{x} + \sigma_{y} } \right)\sin \varphi $$

where $R$ is the radius of the Mohr stress circle.

9 $$ R = \left[ {\frac{1}{4}\left( {\sigma_{x} - \sigma_{y} } \right)^{2} + \tau_{xy}^{2} } \right]^{\frac{1}{2}} $$

The Mohr–Coulomb yield condition can also be expressed in terms of principal stresses $\sigma_{1}$ and $\sigma_{3}$ as follows:

10 $$ \frac{1}{2}\left( {\sigma_{1} - \sigma_{2} } \right) = C\cos \varphi + \frac{1}{2}\left( {\sigma_{1} + \sigma_{3} } \right)\sin \varphi $$

Or

11 $$ \sigma_{1} (1 - \sin \varphi ) - \sigma_{3} (1 + \sin \varphi ) - 2C\cos \varphi = 0 $$

#### Material parameters

The pile model is a linear elastic model, and the soil model is the Mohr–Coulomb model built in ABAQUS, and their detailed parameters are shown in Table 3.

In order to describe the pile-soil contact behavior, the contact properties need to be defined. The shear force of the tangential behavior is proportional to the friction coefficient and a penalty function is used as the friction formula. Here, the friction coefficient of the pile-soil is 0.35.

#### Setting of forces and boundaries

**Figure 5:**
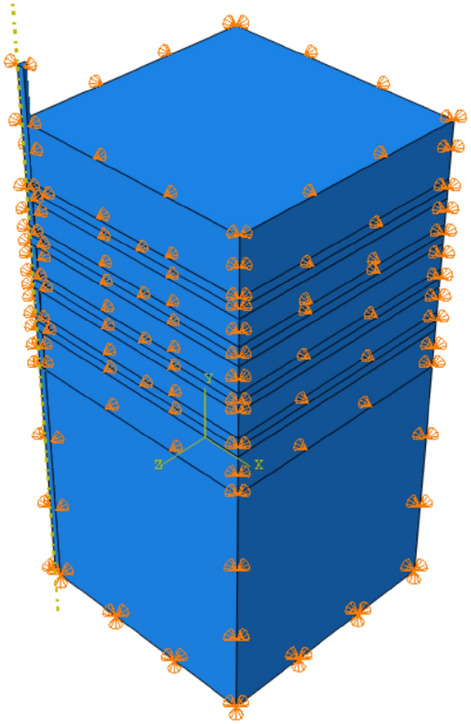
Setting of pile-soil model boundary (Abaqus v6.3).

The load is applied according to the relevant code, using the graded loading method, and ten analysis steps are established in ABAQUS. 200 N is applied to the top of the pile in each analysis step in the form of pressure, considering that the concentrated load will cause non-convergence of the results. All fixed constraints are chosen at the bottom of the soil model, and the side is used to constrain the displacement of the soil in the horizontal direction, as shown in Fig. 5.

#### Division of the grid

**Figure 6:**
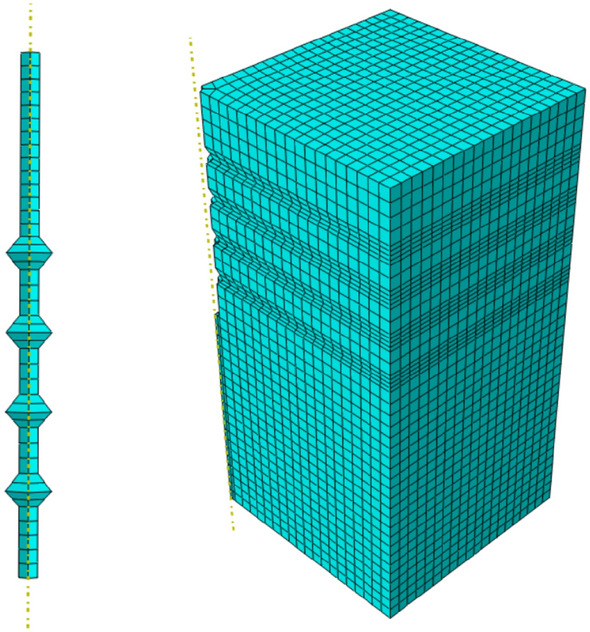
Pile and soil model meshing situation (Abaqus v6.3).

The eight-node hexahedral linear reduction cell C3D8R is used for the pile and soil model, the use of such elements has the following advantages: (1) Shear self-locking is less likely to occur under bending loads. (2) The result of solving for displacement is more accurate. (3) The accuracy of the analysis will not be affected too much when the mesh has twisted deformation. The division of the pile and soil model mesh is shown in Fig. 6.

### Analysis of orthogonal test results

#### Compressive performance orthogonal test

**Figure 7:**
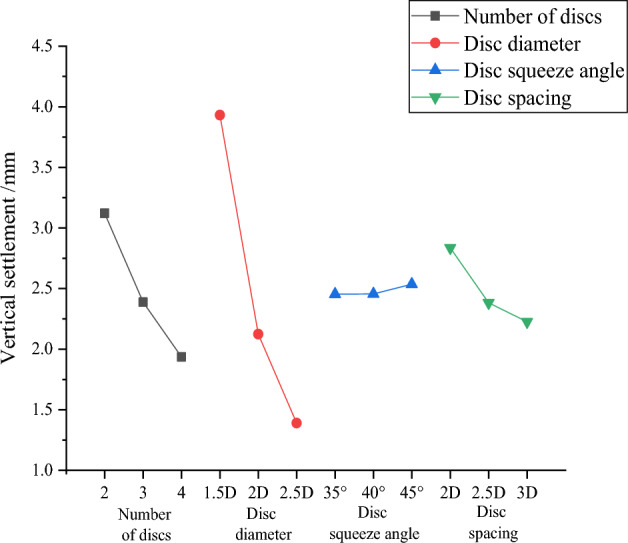
Curve of compressive performance effect.

Numerical simulation obtained 9 groups of simulation results, the 9 groups of simulation results are recorded into the orthogonal test table for range analysis, and the mean value and range corresponding to each level are obtained, as shown in Table 4, and Fig. 7 compressive load bearing performance effect graph is drawn for visual analysis of the calculated results.

**Table 4:** Means and ranges of compressive performance.

Test numbers	Number of discs	Disc diameter	Disc squeeze angle	Disc spacing	Vertical settlement/mm
1	2	1.5D	35°	2D	4.8973
2	2	2D	40°	2.5D	2.6372
3	2	2.5D	45°	3D	1.8271
4	3	1.5D	40°	3D	3.5574
5	3	2D	45°	2D	2.4389
6	3	2.5D	35°	2.5D	1.1712
7	4	1.5D	45°	2.5D	3.3416
8	4	2D	35°	3D	1.2945
9	4	2.5D	40°	2D	1.1726
K1	3.121	3.932	2.454	2.836	
K2	2.389	2.124	2.456	2.383	
K3	1.936	1.390	2.536	2.226	
R	1.185	2.542	0.082	0.610

Based on the means and ranges corresponding to each level, the optimal level corresponding to the minimum value of vertical settlement under each optimization factor is obtained, and the optimal design parameters of the squeezed branch pile under the evaluation index of compressive performance are obtained. Where the larger R is, the more important the factor is.

According to Table 5 and Fig. 7, the order of influence of various factors on the vertical settlement of the squeezed branch pile is from the largest to the smallest: disc diameter, number of discs, disc spacing, and disc squeeze angle.

**Table 5:** Load settlement data.

Pile type	DX1	DX2	DX3	DX4	DX5	DX6	DX7	DX8	DX9	DX10
Q/N	s/mm									
200	0.0486	0.0303	0.0232	0.0286	0.0248	0.0167	0.0250	0.0639	0.0162	0.0139
400	0.1826	0.0752	0.0521	0.0596	0.0577	0.0379	0.0526	0.0835	0.0327	0.0277
600	0.4700	0.2946	0.1242	0.2416	0.2114	0.0939	0.1834	0.1241	0.0664	0.0501
800	0.8303	0.4677	0.3240	0.4637	0.3891	0.1921	0.3830	0.2092	0.1738	0.0882
1000	1.3931	0.8113	0.4490	0.8812	0.7084	0.2964	0.7390	0.3326	0.2695	0.1984
1200	2.0083	1.0888	0.7030	1.2958	0.9599	0.4432	1.1555	0.4837	0.4310	0.3013
1400	2.6550	1.4678	0.9047	1.8463	1.3324	0.5815	1.6775	0.6712	0.5604	0.4272
1600	3.2755	1.8170	1.2076	2.3741	1.6553	0.7861	2.1977	0.8596	0.7836	0.5500
1800	3.9762	2.2389	1.4705	2.9819	2.0656	0.9487	2.7867	1.0759	0.9273	0.7096
2000	4.8973	2.6372	1.8271	3.5574	2.4389	1.1712	3.3416	1.2945	1.1726	0.8527

Through orthogonal test analysis, we can get the optimal combination of factors and levels, i.e., number of discs is 4, disc diameter is 2.5D, disc squeeze angle is 35°, and disc spacing is 3D, which is the best way to reduce the vertical settlement of the pile. The aforementioned values of each level parameter are recorded as the optimal combination of parameters DX10 under the evaluation index of compressive performance.

The same loading process as the first nine groups (DX1-DX9) is carried out for DX10, and vertical compressive performance tests are conducted to obtain load settlement data, which are compared with the first nine groups and compiled to produce Table 5.

**Figure 8:**
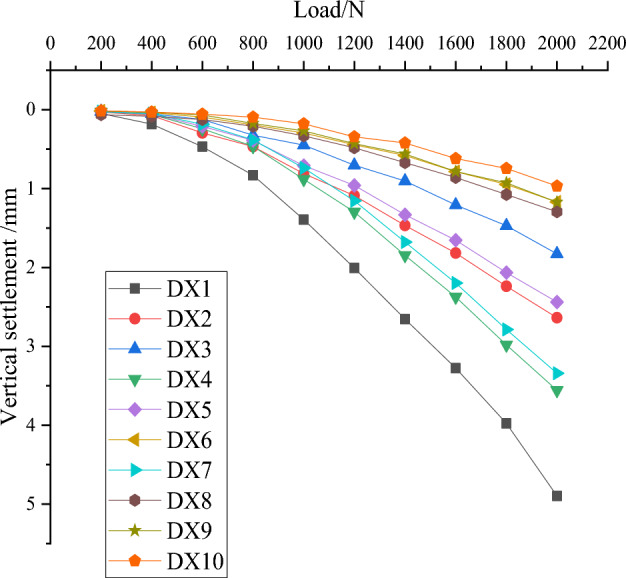
Load settlement curve.

Ten load settlement curves can be drawn based on the ten sets of load settlement data in Table 5, as shown in Fig. 8.

The comprehensive analysis of Table 5 and Fig. 8 concludes that the optimal combination (DX10) has the smallest settlement value of the squeezed branch pile under the same loading conditions and possesses the optimal vertical compressive performance, which further verifies the correctness and scientificity of this orthogonal test.

#### Compressive economic efficiency orthogonal test

Add the linear function y = a in Fig. 8, a being the settlement value corresponding to the optimal combination DX10 when subjected to the maximum load.

**Figure 9:**
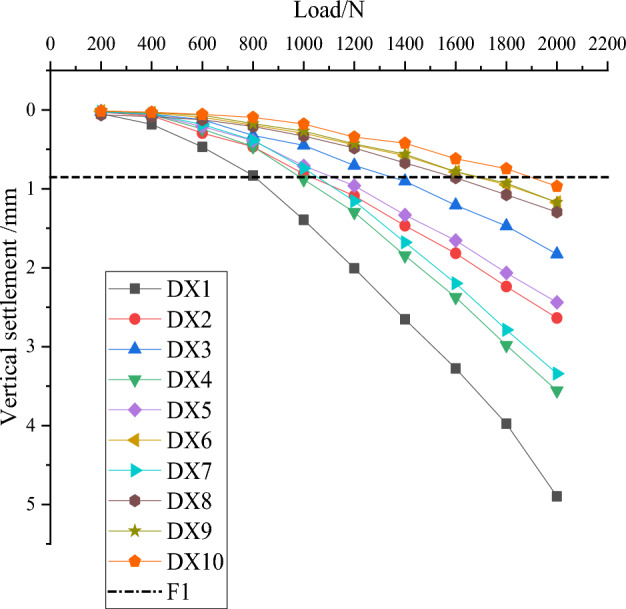
Determine the ultimate bearing capacity of each pile.

In this chapter, a is used as the criterion for determining the ultimate bearing capacity of squeezed branch pile. From Table 5 and Fig. 8, it is known that a is the settlement value of 0.8527mm produced by the DX10 model under 2000N load, therefore, a linear function y = 0.8527 is added, as shown in Fig. 9.

The cross coordinates of the intersection of the linear function y = a and the other nine load settlement curves are used as the ultimate bearing capacity of the other nine squeezed branch piles. According to the ultimate bearing capacity and volume of the other nine squeezed branch piles, the unit volume bearing capacity provided by the unit volume of the nine squeezed branch piles is obtained respectively, and the unit volume bearing capacity values of each pile are shown in Table 6.

**Table 6:** Bearing capacity per unit volume of squeezed branch piles.

Pile type	Ultimate bearing capacity/N	Volume/cm^3^	Compressive bearing capacity per unit volume/(N/cm^3^)
DX1	807.964	432.78	1.866
DX2	1029.847	471.56	2.183
DX3	1348.403	567.25	2.377
DX4	986.340	439.68	2.243
DX5	1114.726	480.57	2.319
DX6	1681.957	574.46	2.927
DX7	1054.590	448.54	2.351
DX8	1592.723	503.31	3.164
DX9	1696.173	664.33	2.553

The unit volume bearing capacity values of nine groups of squeezed branch piles from DX1-DX9 are filled into the orthogonal table for the calculation of economic efficiency, and the mean and range of compressive economic efficiency are collated to obtain Table 7.

**Table 7:** Mean and range of compressive economic efficiency.

Factor	Number of discs	Disc diameter	Disc squeeze angle	Disc spacing
K1	2.143	2.154	2.653	2.247
K2	2.497	2.556	2.327	2.488
K3	2.690	2.619	2.349	2.595
R	0.547	0.465	0.326	0.348

**Figure 10:**
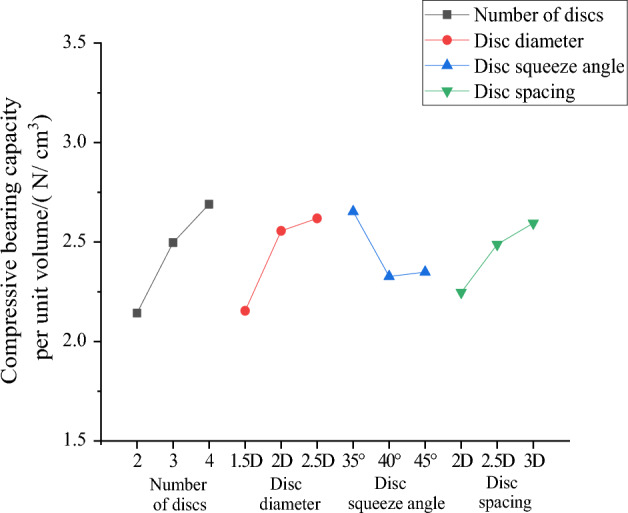
Curve of compressive economic efficiency effect.

According to Table 7, the order of influence of various factors on the bearing capacity provided by the unit volume of the squeezed branch pile is obtained, and the curve of the economic efficiency effect of compressive resistance is drawn in Fig. 10. The analysis of the calculation results shows that the order of influence of the four factors is from the largest to the smallest: number of discs, disc diameter, disc spacing, disc squeeze angle.

The optimized design parameters of economic efficiency can be obtained through Table 7 and Fig. 10, namely, the number of discs is 4, disc diameter is 2.5D, disc squeeze angle is 35°, disc spacing is 3D, and the optimized design parameter is the squeezed branch pile with the largest bearing capacity per unit volume, i.e., the squeezed branch pile disc pile with the best economic efficiency of compressive resistance. It is further found that this combination has the same parameters as the combination with the best compressive performance (DX10), and it can be concluded that DX10 has the best combination of factors in terms of both compressive performance and compressive economic efficiency.

The bearing capacity per unit volume provided by DX10 model pile is shown in Table 8, and the comparison between Tables 6 and 8 shows that the squeezed branch pile DX10 is the model pile with the best compressive economic efficiency of the squeezed branch pile.

**Table 8:** Bearing capacity per unit volume of DX11.

Pile type	Ultimate bearing capacity/N	Volume/cm^3^	Compressive bearing capacity per unit volume/(N/cm^3^)
DX10	2000	624.51	3.2025

When the parameters of the optimal combination of compressive performance and the optimal combination of compressive economic efficiency are consistent, they are taken as the optimal design parameters. When the two aspects do not agree, the designer needs to determine the actual requirements and select the side with the higher weight as the optimal design parameter.

#### Optimal test combination of axial force and load sharing analysis

The axial force analysis of the squeezed branch pile designed according to the optimal design parameters is carried out to provide guidance for the actual installation of the squeezed branch piles in the project.

The main difference between the squeezed branch pile and the straight pile is that the disc of the squeezed branch pile will resist the external force together with the pile body under the external load, providing additional bearing capacity. In order to investigate the specific load-bearing law of the optimal combination of squeezed branch piles, the aforementioned combination DX10, which has the optimal compressive performance and compressive economic efficiency, is subjected to axial force analysis to obtain the load transfer mechanism of DX10.

**Figure 11:**
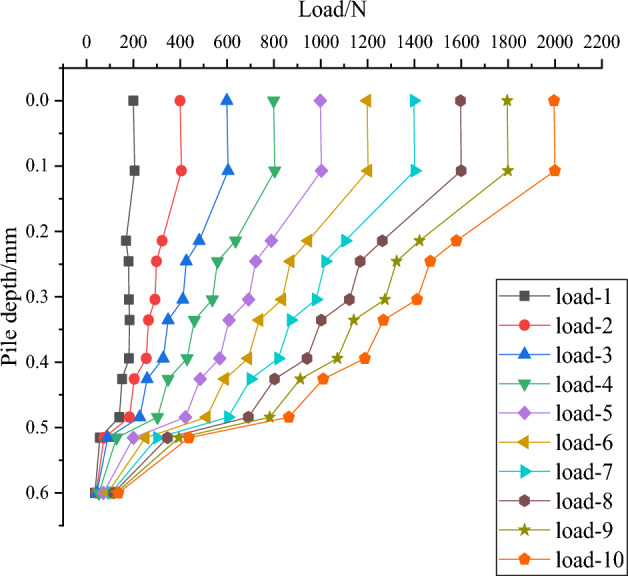
Axial force transfer curve of DX10.

As shown in Fig. 11, the axial force transfer curve of DX10 clearly shows that the axial force of the squeezed branch pile decreases with increasing depth, which is similar to the performance of straight piles. However, the difference is that in the squeezed branch pile, there is a significant change in axial force at the interface between the top and bottom of the disc (where load-1 to load-10 are external loads in 200N increments), and this reduced axial force is carried by the discs and transferred to the soil below discs. As the load at the top of the pile increases, the reduction of the pile axial force at the disc becomes larger and larger. This is the characteristic of the compressive performance of the squeezed branch pile and the reason for its high bearing capacity.

**Figure 12:**
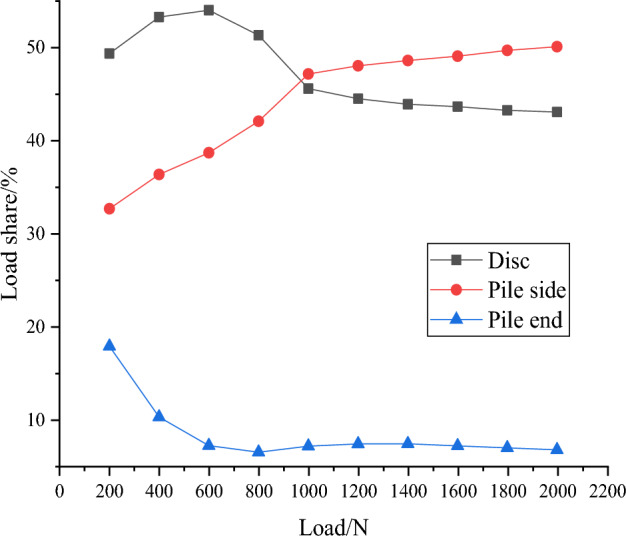
Load sharing curve of DX10.

The axial force of each part of the pile is extracted in the software ABAQUS to produce Fig. 12, the load sharing curve of DX10. The analysis shows that in the whole loading stage, the load shared by discs has been 43–55%, which means that the axial force shared by discs accounts for a large part of the whole pile. Among all discs, the one closest to the pile end has the largest bearing ratio, so its location selection is especially important, and in engineering practice it should be preferred to be placed in the high quality soil layer to provide greater bearing capacity.

The soil below the discs is squeezed, which makes the lateral frictional resistance of the pile between discs much higher. The axial force borne by the lateral friction resistance increases with the external load, from 32% all the way up to about 50%, which plays an important role in improving the single pile bearing capacity.

Of all discs, the one closest to the pile end reduces the axial force the most. At the same time, as each disc shares some of the pile axial force, it ultimately leads to an inevitable reduction in pile end resistance. Therefore, under the same load conditions, the pile end resistance of the squeezed branch pile is inevitably smaller than that of the ordinary straight pile, which is intuitively reflected by the fact that the pile top settlement of the squeezed branch pile is smaller than that of the ordinary straight pile.

#### Analysis of soil displacement and stress fields around piles for optimal test combination

**Figure 13:**
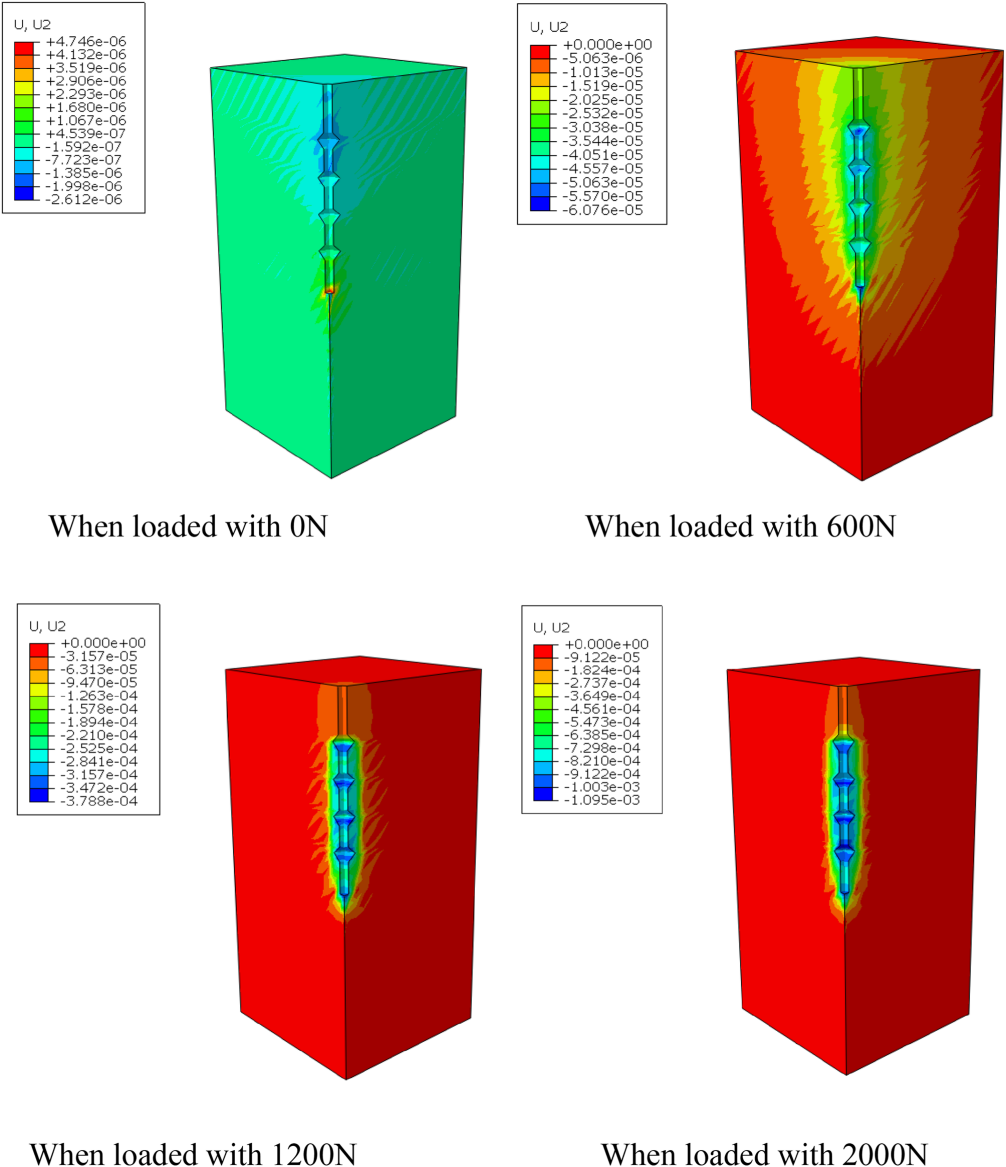
Variation of displacement field of DX10 during 2000N loading (Abaqus v6.3).

**Figure 14:**
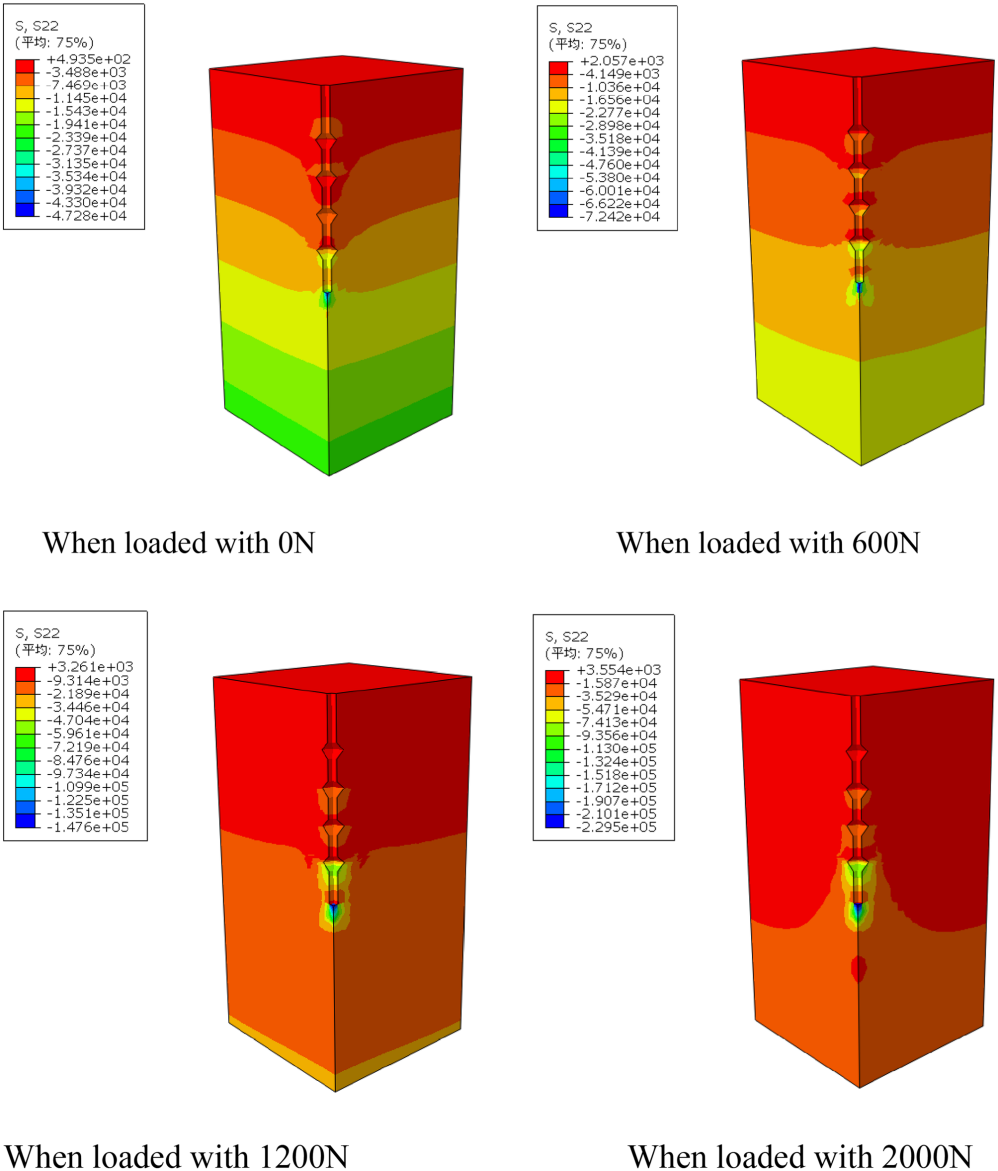
Variation of stress field of DX10 during 2000N loading (Abaqus v6.3).

By using the frame selector function in the visualization module of the finite element software, the variation law of displacement field and stress field of the soil around the pile during the loading process is further analyzed, and the variation of displacement field and stress field is shown in Figs. 13 and 14.

By analyzing Fig. 13, it can be found that the variation of soil displacement around the pile is concentrated near the pile body. With the increase of external load, the soil near the discs moves much more compared with other parts of the soil, indicating that the soil on the lower side of the disc is sufficiently compressed by the load transferred from the disc to provide a larger bearing capacity.

It can be seen from the graph of the variation of soil stress field around the pile that the soil on the lower side of the pile end and the soil on the lower side of the discs are the main areas of compressive stress concentration, which is due to the fact that the discs and the pile end of the squeezed branch pile bear more load. The compressive stress in the disc closest to the pile end is the largest, and the stress is distributed in a circular pattern, the further away from the pile the smaller the stress is, while the soil on the upper side of the disc is subject to tensile stress due to slip with the pile.

**Figure 15:**
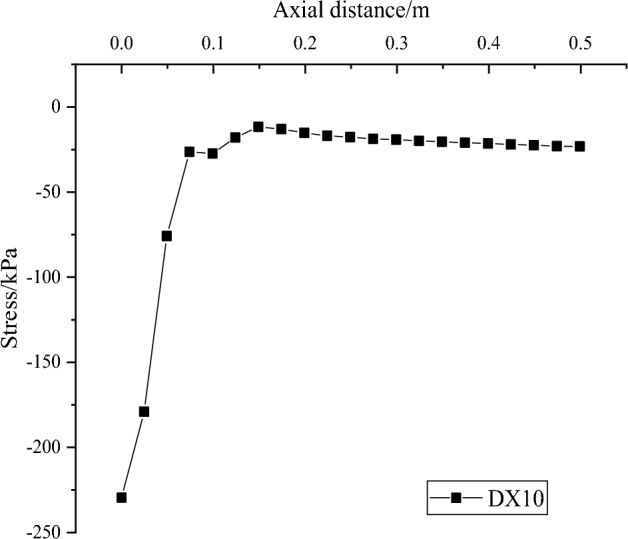
Distribution of soil stress along the axial direction at the pile end of DX10.

The distribution of soil stress along the axial direction at the pile end of DX10 is obtained by selecting a number of node numbers, creating a path distributed along the axial direction of the squeezed branch pile, setting the output variable to S22, the vertical compressive stress, for the last analysis step, and plotting the curve. Observing Fig. 15, it can be found that the main compressive stresses in the soil at the pile end of the squeezed branch pile are distributed within a range of 0.1m from the axial distance of the pile end, and the stresses outside the range will gradually tend to a stable value. It means that the load at the top of the pile is transferred to the pile end along the pile body, and then the pile end is transferred to the soil below the pile end, and the stresses diffused into the soil will only exist at a closer distance from the pile end, and cannot be transferred to a farther distance.

## Finite element analysis of uplift and horizontal resistance capabilities of squeezed branch piles

### Finite element analysis of the uplift resistance capabilities of squeezed branch piles

#### Uplift performance orthogonal test

Squeezed branch piles are widely used in practical engineering applications and scientific research as compression-bearing structures, but the research and application in uplift resistance are rare. In large underground buildings, marine terminals, suspension bridges, etc., the squeezed branch pile as a uplift-resistant structure greatly increases the contact area between the pile and the soil compared to the ordinary straight pile with less uplift resistance, thus enhancing the uplift resistance.

Although the compressive and uplift resistance of the squeezed branch piles are both part of the vertical bearing performance of the piles, different structural requirements, such as the number and spacing of the discs, need to be considered due to the different stressing methods. This chapter analyzes the major and minor factors affecting the uplift resistance of the squeezed branch pile by simulating the nine working conditions designed by orthogonal tests with finite element software, and finds out the optimal or better combination of uplift resistance.

The finite element model is basically the same as that in Chapter 3, but the main difference lies in the direction and magnitude of the applied force. In this chapter, the force applied on the squeezed branch pile is the vertical upward uplift force, and the uplift force is 100N per stage, totaling ten stages.

**Figure 16:**
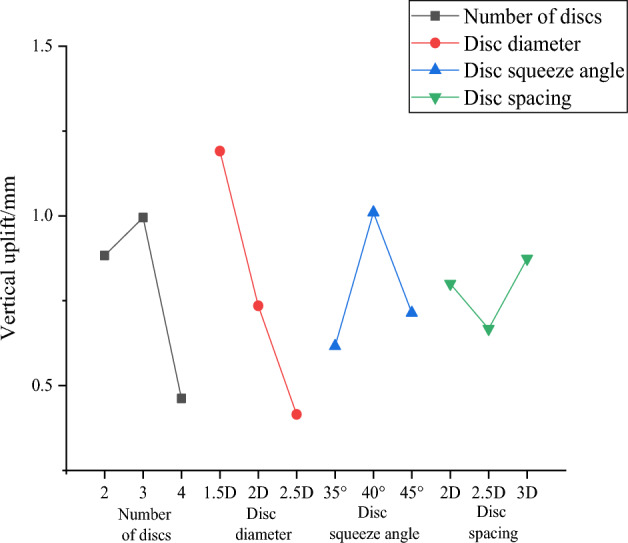
Curve of uplift performance effect.

Numerical simulations are performed for nine working conditions, respectively, and the nine sets of simulation results obtained are filled in the orthogonal test Table 9 with the examined upward displacement for range analysis. And then, the calculated mean and range are plotted in Fig. 16 for the effect of uplift performance.

**Table 9:** Mean and range of uplift performance.

Test numbers	Number of discs	Disc diameter	Disc squeeze angle	Dis spacing	Vertical uplift/mm
1	2	1.5D	35°	2D	1.1499
2	2	2D	40°	2.5D	0.9540
3	2	2.5D	45°	3D	0.5459
4	3	1.5D	40°	3D	1.7292
5	3	2D	45°	2D	0.9034
6	3	2.5D	35°	2.5D	0.3518
7	4	1.5D	45°	2.5D	0.6937
8	4	2D	35°	3D	0.3466
9	4	2.5D	40°	2D	0.3458
K1	0.883	1.191	0.616	0.800	
K2	0.995	0.735	1.010	0.667	
K3	0.462	0.415	0.714	0.874	
R	0.533	0.776	0.394	0.207

Combined with Table 9 and Fig. 16, the influence of various factors on the vertical upward displacement of the squeezed branch pile is in the following order: disc diameter, number of discs, disc squeeze angle, and disc spacing. The optimal combination of factor parameters is disc diameter of 2.5D, discs of 4, disc squeeze angle of 35°, and disc spacing of 2.5D, which is the optimal combination to reduce the vertical upward displacement of the squeezed branch pile, and this combination is recorded as DX11.

Since the optimal combination DX11 is not in the initial working condition, it is necessary to build a model based on the specific parameters of the optimal combination, load the DX11 simulation, compare it with the nine sets of initial working conditions, and compile and produce Table 10.

**Table 10:** Uplift load displacement data.

Pile type	DX1	DX2	DX3	DX4	DX5	DX6	DX7	DX8	DX9	DX11
Q/N	s/mm									
100	0.0146	0.0116	0.0091	0.0110	0.0093	0.0071	0.0091	0.0067	0.0062	0.0065
200	0.0331	0.0262	0.0199	0.0252	0.0204	0.0150	0.0197	0.0141	0.0137	0.0137
300	0.1070	0.0513	0.0336	0.0481	0.0342	0.0235	0.0320	0.0219	0.0215	0.0211
400	0.1763	0.1015	0.0595	0.0879	0.0726	0.0333	0.0551	0.0304	0.0305	0.0293
500	0.3030	0.1663	0.0866	0.1806	0.1063	0.0473	0.0805	0.0405	0.0445	0.0388
600	0.4166	0.2824	0.1545	0.3133	0.2216	0.0885	0.1804	0.0568	0.0641	0.0527
700	0.5887	0.3882	0.1884	0.5546	0.3306	0.1325	0.2394	0.0846	0.1159	0.0795
800	0.7347	0.5859	0.3245	0.8156	0.4948	0.1967	0.3670	0.1391	0.1568	0.1471
900	0.9629	0.7225	0.3931	1.2409	0.6975	0.2671	0.4907	0.2204	0.2368	0.1870
1000	1.1500	0.9540	0.5460	1.7292	0.9035	0.3519	0.6938	0.3466	0.3459	0.3038

**Figure 17:**
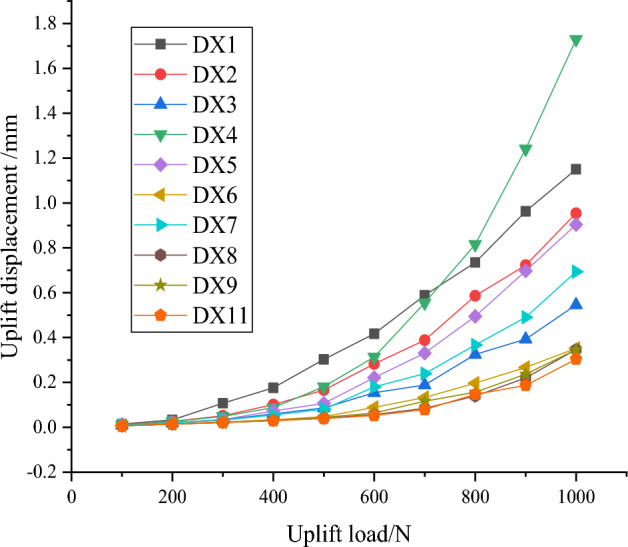
Load displacement curve.

According to the ten sets of uplift load displacement data in Table 10, Fig. 17 is plotted. comprehensive analysis of Table 10 and Fig. 17 shows that the optimal combination of uplift resistance DX11 has the best uplift resistance, which further verifies the correctness of this orthogonal test.

#### Uplift economic efficiency orthogonal test

DX1-DX9 uplift ultimate bearing capacity is obtained in the same way as compressive ultimate bearing capacity, which is not repeated here, and the uplift bearing capacity per unit volume of each working condition is calculated by volume, as shown in Table 11.

**Table 11:** Bearing capacity provided by unit volume of squeezed branch pile.

Pile type	Ultimate bearing capacity/N	Volume/cm^3^	Uplift bearing capacity per unit volume/( N/ cm3)
DX1	500.717	432.78	1.1569
DX2	620.264	471.56	1.3153
DX3	784.807	567.25	1.3835
DX4	592.842	439.68	1.3483
DX5	675.422	480.57	1.4054
DX6	943.270	574.46	1.6420
DX7	750.453	448.54	1.6731
DX8	966.081	503.31	1.9194
DX9	961.417	664.33	1.4472

**Figure 18:**
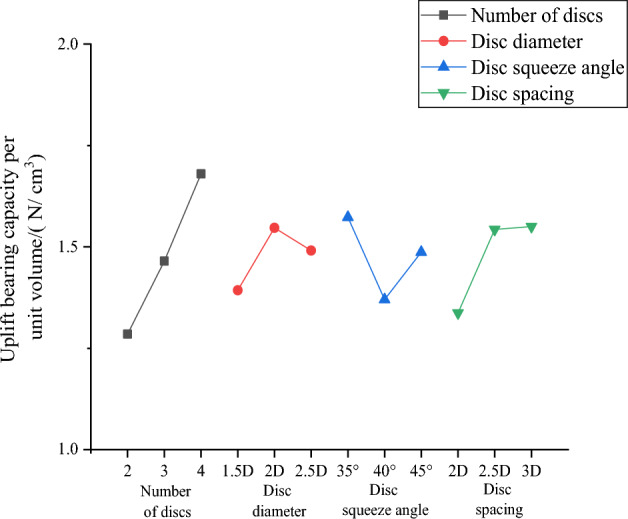
Curve of uplift economic efficiency effect.

The uplift bearing capacity per unit volume of the nine working conditions is filled into the orthogonal table, and Table 12 is obtained through the range analysis of the orthogonal table. Then according to Table 12, Fig. 18 is drawn to visualize the differences of the influencing factors.

**Table 12:** Mean and range of uplift economic efficiency.

Factor	Number of discs	Disc diameter	Disc squeeze angle	Disc spacing
K1	1.285	1.393	1.573	1.337
K2	1.465	1.547	1.370	1.543
K3	1.680	1.491	1.487	1.550
R	0.395	0.154	0.203	0.213

According to the range analysis, the order of influence of the four factors is from the largest to the smallest: number of discs, disc spacing, disc squeeze angle, and disc diameter. Based on the mean value of the four factors, it can be inferred that the parameters of the optimal combination are 4 discs, 2D disc diameter, 35° disc squeeze angle and 3D disc spacing, which are identical to the parameters of the initial combination DX8, indicating that DX8 is the optimal combination for the uplift economic efficiency.

#### Optimal test combination of axial force and load sharing analysis

Axial force analysis of DX11, the optimal uplift resistance combination that can provide the maximum uplift resistance, is performed to explore the underlying logic of its uplift resistance and to provide a qualitative analysis for the study of uplift resistant squeezed branch piles.

**Figure 19:**
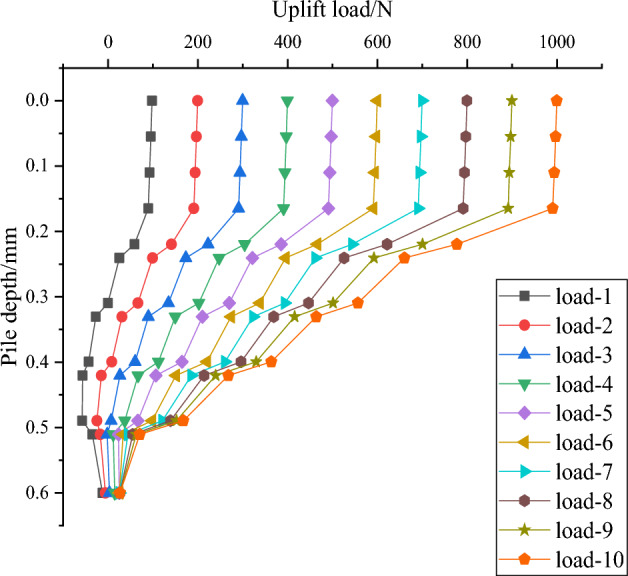
Axial force transfer curve of DX11.

The uplift resistance axial force transfer curve of DX11 (Fig. 19) is similar to the compressive axial force transfer curve in that the axial force of the pile undergoes a significant reduction at the upper and lower sections of the discs, and these reduced axial forces are shared by the discs and provide the uplift resistance by compressing the soil above the discs.

**Figure 20:**
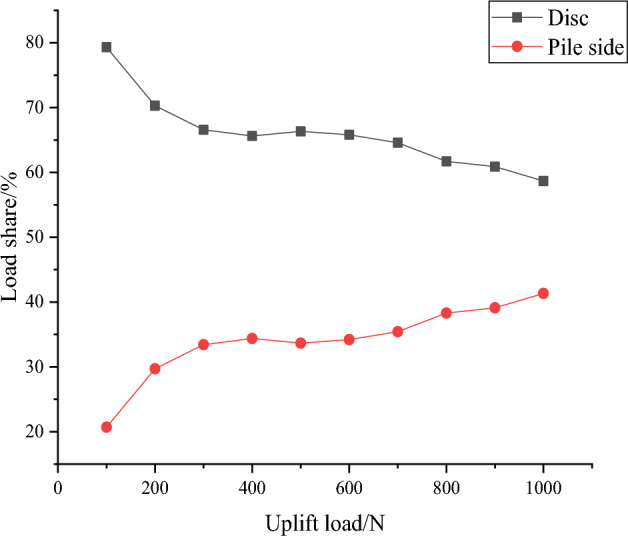
Load sharing curve of DX11.

The uplift resistance of the squeezed branch pile mainly comes from two aspects: the disc end resistance and the pile side frictional resistance, where the disc end resistance bears 58.6–79.3% of the total uplift load, while the pile side frictional resistance bears less, accounting for 20.7–41.3%. Through Fig. 20, it is obvious that the percentage of the load borne by the discs gradually decreases with the increase of the uplift load, while the pile side is exactly the opposite.

Among the four discs, the uppermost one provides the highest load carrying capacity in all bearing stages, ranging from 28.6% to 56.6% in different stages.

#### Analysis of soil displacement and stress fields around piles for optimal test combination

**Figure 21:**
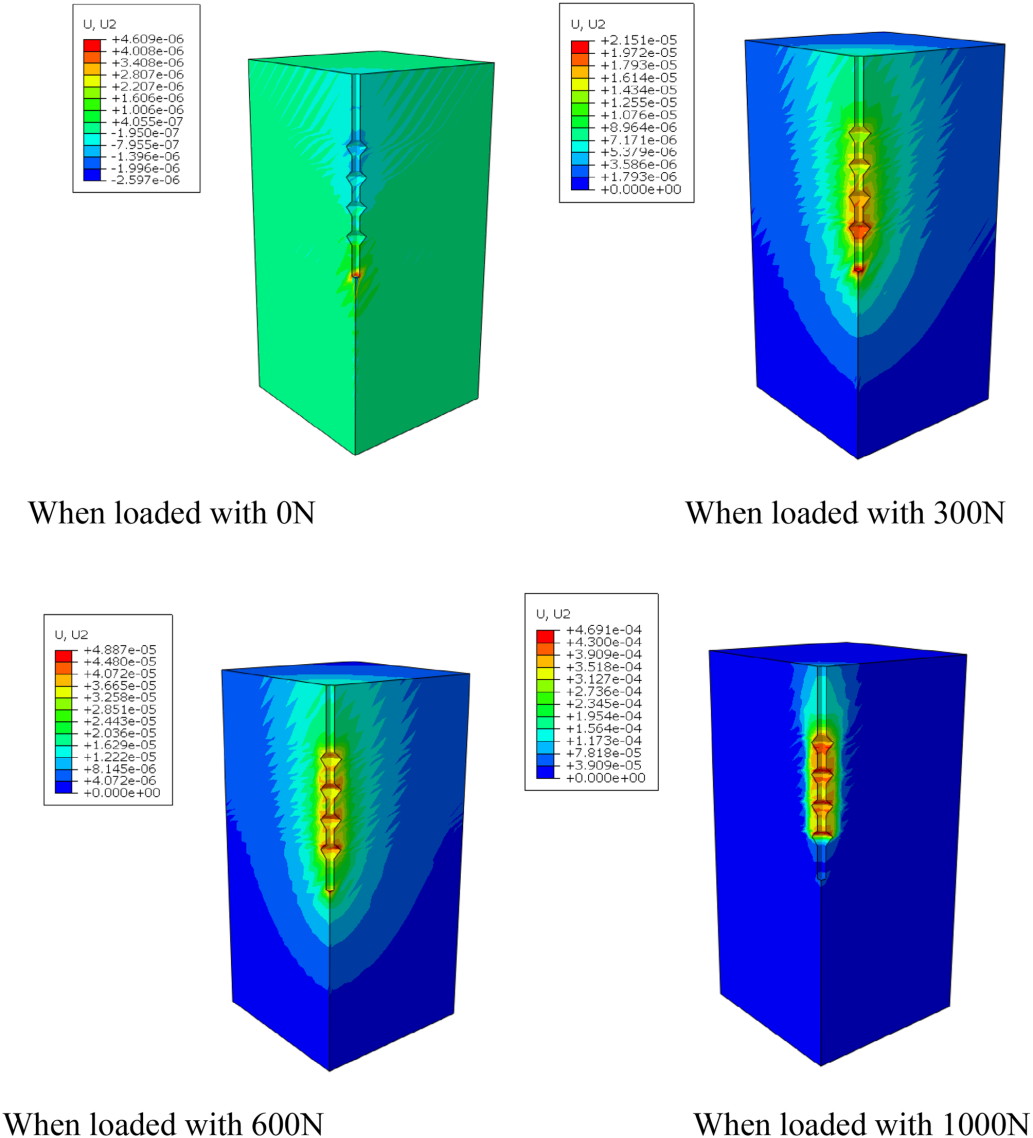
Variation of displacement field of DX11 during 1000N loading (Abaqus v6.3).

**Figure 22:**
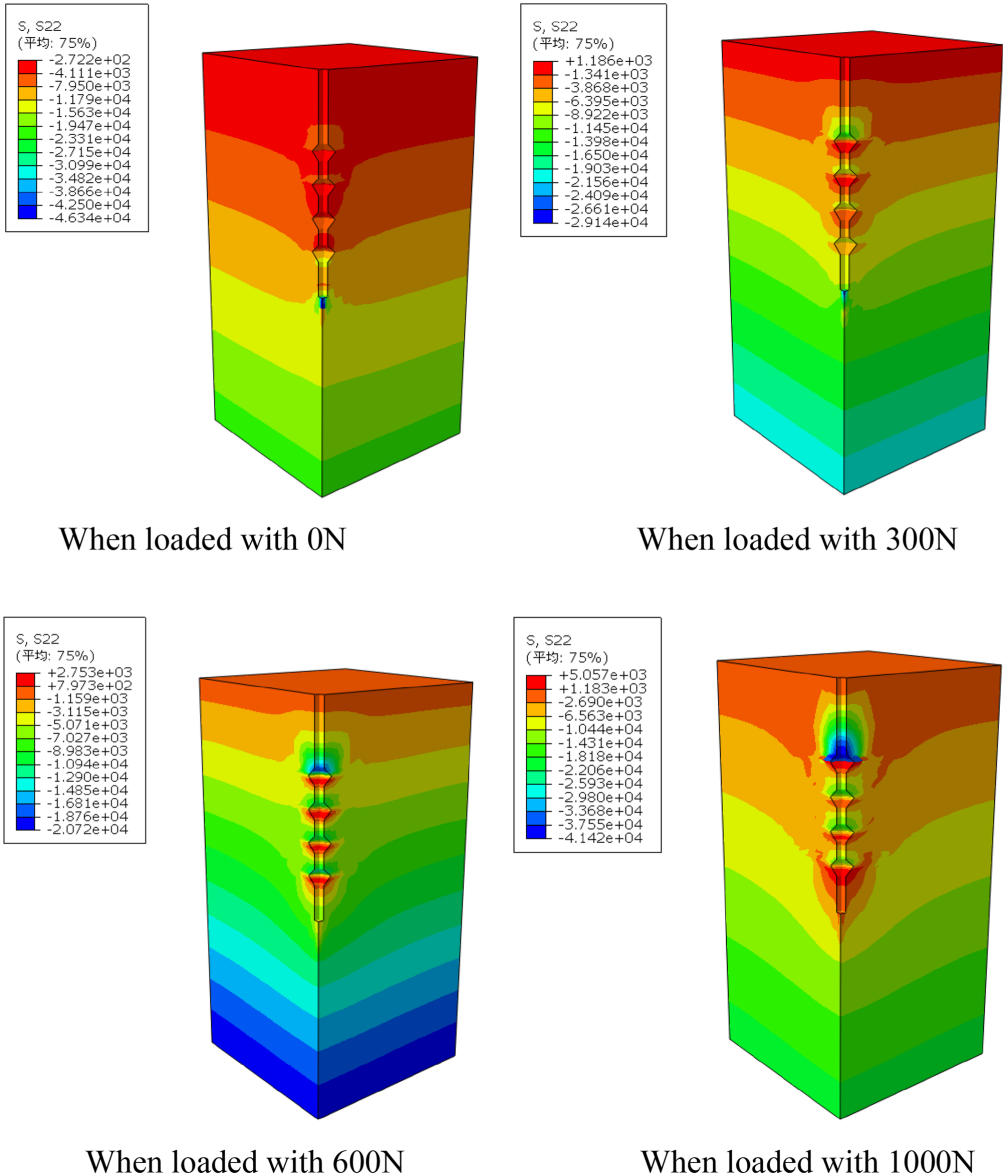
Variation of stress field of DX11 during 1000N loading (Abaqus v6.3).

By observing the soil displacement field Fig. 21 and stress field Fig. 22 of DX11, some patterns can be found. At the early stage of loading, the soil displacement near the lower side of the discs changes greatly, while the soil displacement near the upper side of the discs does not change significantly. As the loading continues, the soil on the upper side of the four discs is repeatedly compressed, resulting in larger displacement, and the displacement of the soil between the discs also increases to a small extent, and the discs make the soil around the pile fully exert the uplift resistance.

The soil on the lower side of the disc is under tension, and the soil on the upper side of the disc is under pressure, with the soil on the upper side of the uppermost disc under the greatest pressure.

### Finite element analysis of horizontal resistance capabilities of squeezed branch piles

#### Model building

**Figure 23:**
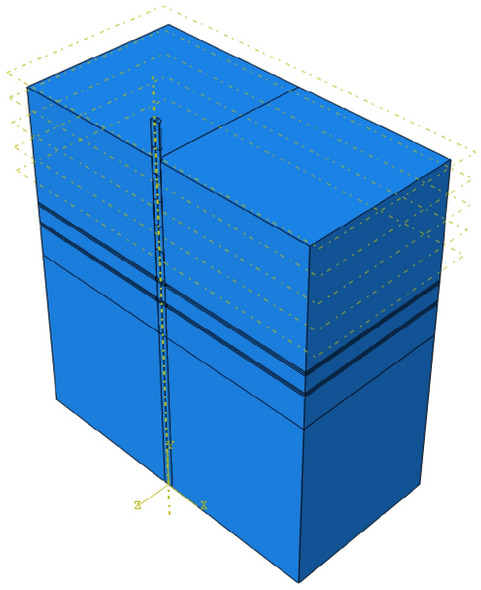
1/2 model (Abaqus v6.3).

For the squeezed branch pile with horizontal load at the top of the pile, half of the pile-soil model along the loading direction is taken for the finite element simulation in consideration of the symmetry of the forces and boundaries when building the model. The material parameters, contact settings and boundary settings are the same as the process of finite element simulation in Chapter 3. To avoid repeated modeling, the 1/4 model above is mirrored by the mirror function to obtain the 1/2 model figure as shown in Fig. 23.

Since the FEM software ABAQUS does not output the bending moment of each section of the support pile by default, it is necessary to modify the inp file to output the internal force of the section before the model calculation. Take the first section of DX1 as an example, execute [Model]/[Edit Keywords] and enter the following statement after the analysis step for loading:

*section print,name = s1,surface = pile-1.s1,axes = local,frequency = 1,update = yes.

,0,1.1,0

,1,1.1,0,,0,1.1,1

Sof,som.

‘name’ in the keyword is the name of the user-specified output identifier, which is set to s1–s13 in this chapter according to the different pile types, where DX1 has eleven output sections. ‘surface’ specifies the faces that have been defined, and each pile type in this chapter has between 10 and 13 different output sections. ‘axes = local’ means local coordinate system. ‘frequency = 1’ means the output frequency is 1. ‘update = yes’ means to update the coordinate system in case of geometric nonlinearity.

The coordinates in the second line of the statement are the shape center coordinates of the complete section, the two sets of coordinates in the third line correspond to points a and b in the figure, and sof in the fourth line denotes the axial force and som denotes the bending moment.

In order to simulate the force situation of the squeezed branch pile under the lateral action such as earthquake, the load type is selected as surface load in the loading analysis step, and then the top plane of the pile is selected as the loading surface, and the loading process is carried out in ten stages with 200N per stage according to the relevant specifications.

#### Analysis of orthogonal test results of horizontal resistance capabilities of squeezed branch piles

**Figure 24:**
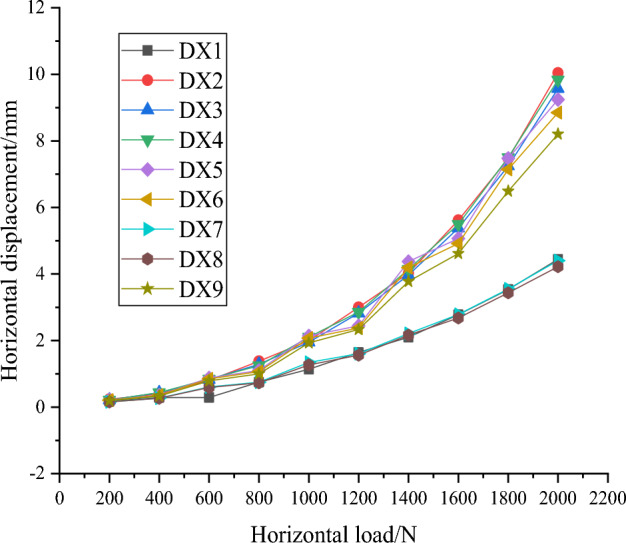
Variation of horizontal displacement of pile top with horizontal load.

**Figure 25:**
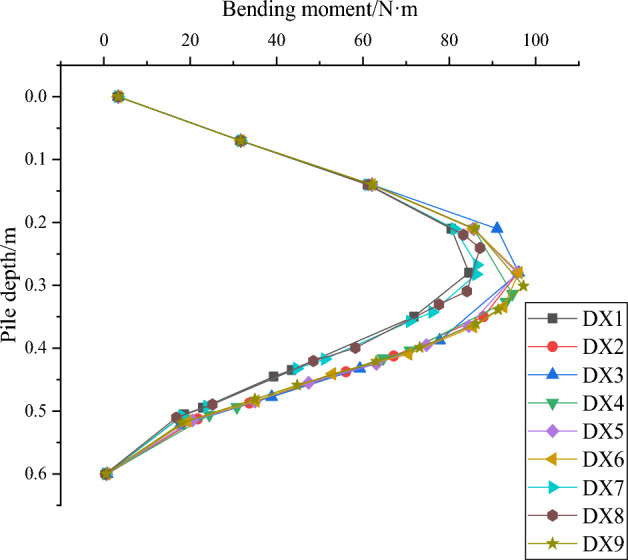
Variation of pile bending moment with pile depth.

In this paper, for the study of horizontal load resistance capabilities of squeezed branch pile, the horizontal displacement of pile top and the maximum bending moment of pile body are the two main research focuses. Firstly, the variation of horizontal displacement of pile top with horizontal load and the variation of pile bending moment with pile depth from nine sets of test results are plotted in Figs. 24 and 25. The results show that the horizontal displacement of the top of the squeezed branch pile increases with the increase of the horizontal load on the top of the pile; the bending moment of the pile increases and then decreases along the top of the pile to the bottom of the pile, and reaches the maximum value at about 0.3m depth of the pile.

**Figure 26:**
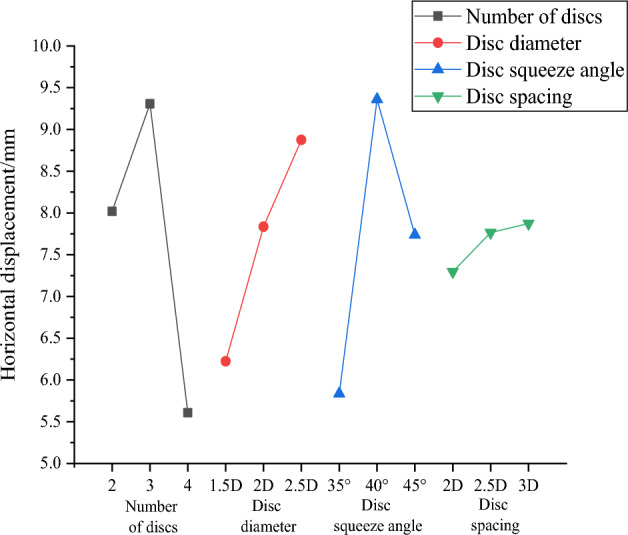
Curve of horizontal displacement of pile top effect top with horizontal load.

**Figure 27:**
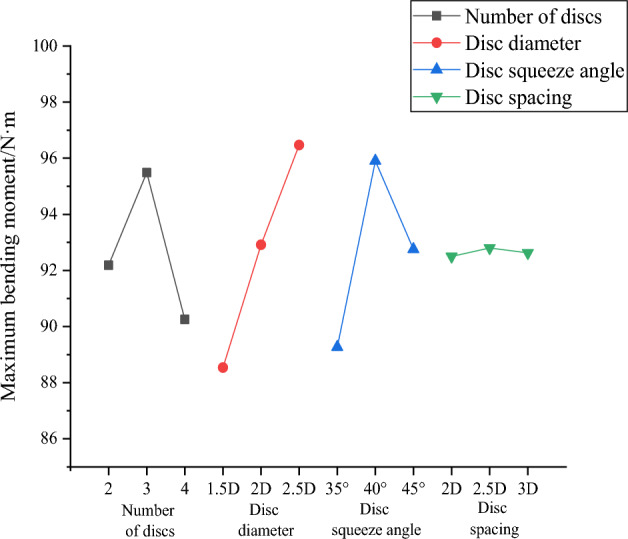
Curve of maximum bending moment of the pile effect.

The maximum horizontal displacement of the pile top and the maximum pile bending moment of each group of working conditions are filled into the orthogonal table to calculate the mean and range to obtain Tables 13 and 14, and then the effect curves are plotted in Figs. 26 and 27. The analysis shows that the order of factors influencing the horizontal displacement of squeezed branch piles are: number of discs, disc squeeze angle, disc diameter and disc spacing in descending order. And the factors influencing the maximum bending moment of the pile body of the squeezed branch pile are, in descending order, disc diameter, disc squeeze angle, number of discs and disc spacing. The two sets of optimization objectives are simultaneously optimized for the number of 4 discs, disc diameter of 1.5 D, disc squeeze angle of 35° and disc spacing of 2D.

**Table 13:** Mean and range of horizontal displacement of pile top.

Factor	Number of discs	Disc diameter	Disc squeeze angle	Disc spacing
K1	8.019	6.224	5.836	7.296
K2	9.307	7.835	9.360	7.765
K3	5.608	8.875	7.738	7.874
R	3.699	2.651	3.524	0.578

**Table 14:** Mean and range of maximum bending moment of the pile.

Factor	Number of discs	Disc diameter	Disc squeeze angle	Disc spacing
K1	92.187	88.540	89.270	92.500
K2	95.487	92.917	95.903	92.800
K3	90.253	96.470	92.753	92.627
R	5.234	7.930	6.633	0.300

**Figure 28:**
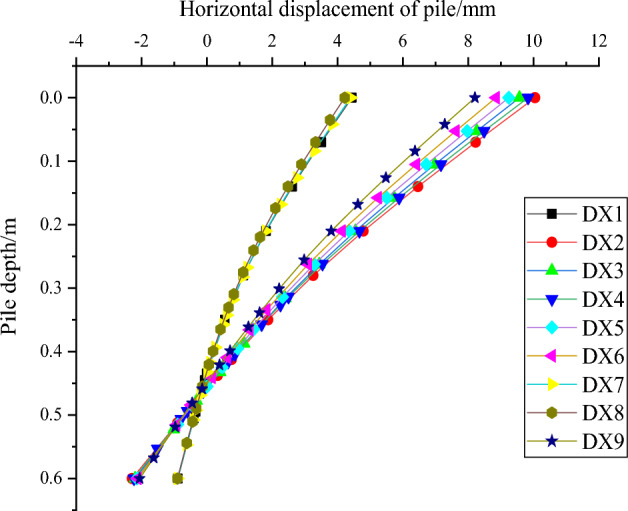
Distribution of horizontal displacement along the pile.

As shown in Fig. 28, the distribution of pile horizontal displacement along the pile body is generally regular, the pile horizontal displacement decreases as the pile depth increases, and the pile horizontal displacement near the end of the pile appears negative, where the displacement in the positive direction of the x-axis of the finite element software is positive, and vice versa is negative.

**Figure 29:**
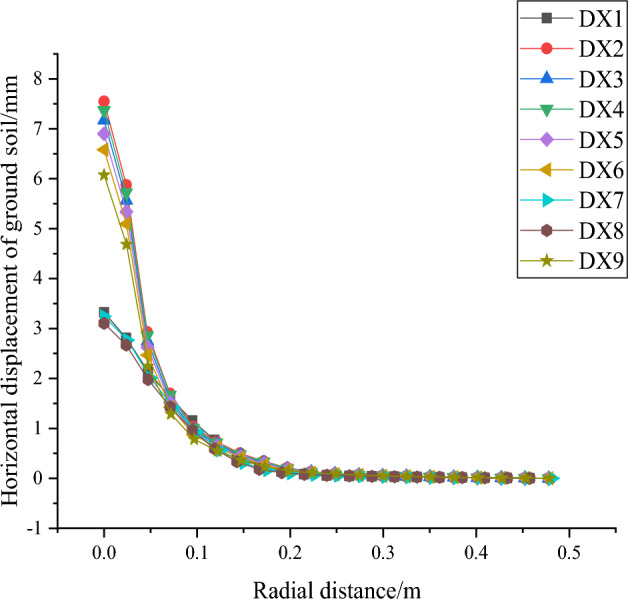
Variation of horizontal displacement of ground soil in front of pile with radial distance.

As shown in Fig. 29, the horizontal displacement of the ground soil in front of the pile (loading direction is the front) decreases with the increase of radial distance from the pile, reaching a radial distance of about 0.3m (10 times the pile diameter) almost remains unchanged, indicating that the influence of the squeezed branch pile on the soil has been negligible at this time.

**Figure 30:**
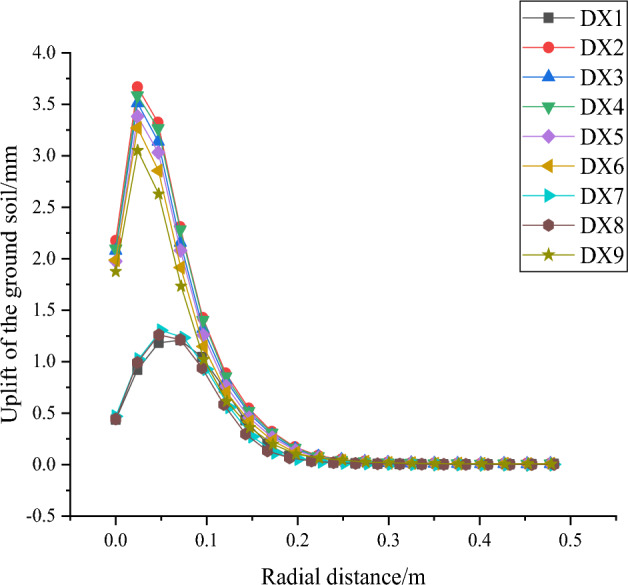
Uplift of the ground soil in front of the pile with radial distance.

As shown in Fig. 30, the uplift of the ground soil in front of the pile increases and then decreases with the increase of the radial distance, and most of the piles reach the maximum value around 0.06m (2 times the pile diameter) of the radial distance, and then slowly decreases and remains unchanged.

**Figure 31:**
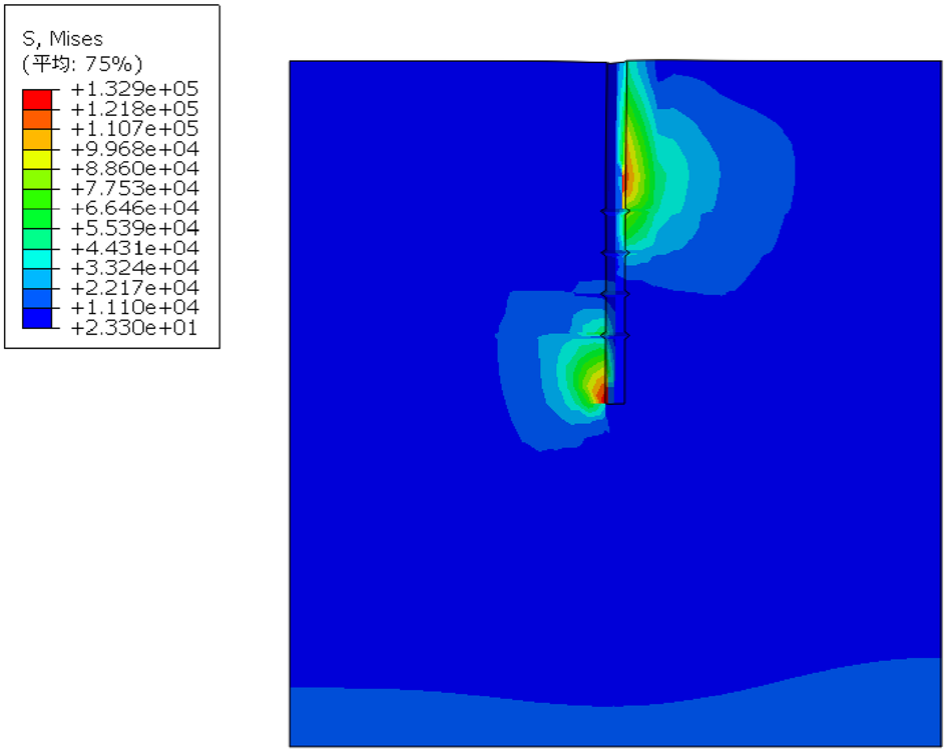
Soil stress field around the pile (Abaqus v6.3).

**Figure 32:**
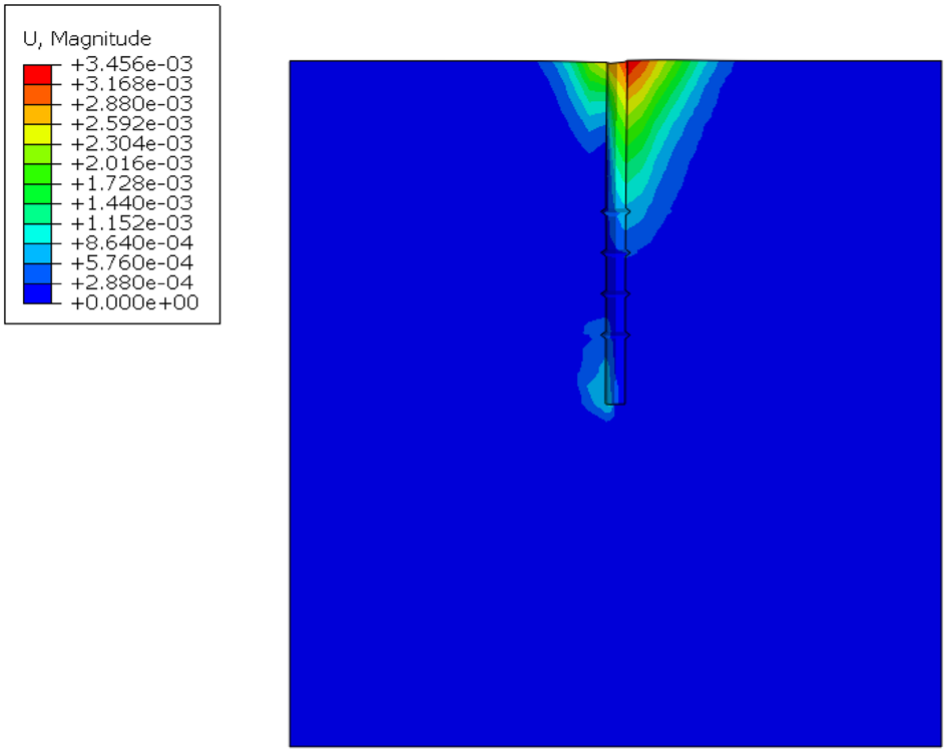
Soil displacement field around the pile (Abaqus v6.3).

By analyzing Fig. 31 the stress field of the soil around the pile for the optimal combination of horizontal bearing performance, it can be found that the maximum stress of the soil appears on the left side of the pile end and the right side of the middle part of the pile; from Fig. 32 the displacement field of the soil around the pile, it can be seen that the displacement change of the soil around the pile is concentrated on the right side of the pile top near the ground, and a small part appears on the left side of the pile end.

## Multi-objective optimization design

The optimal or better combination obtained from a single-objective orthogonal test is the one that makes the performance of an individual optimal or better under some defined rules, while there may be conflicting situations between multiple objectives when multiple metrics are considered at the same time. To achieve balance and coordination among different objectives, this chapter optimizes multiple objectives through multi-objective optimization design, aiming to find a set of optimal solutions that satisfy the balance and contradiction between various objectives and constraints.

Multi-objective optimal design is widely used in engineering, economy, environment and other fields, especially in the design and decision-making process of complex systems, which can help decision makers to grasp the complexity of things, so as to improve the quality and efficiency of decision-making. The analysis methods of multi-objective orthogonal test results are commonly used in "Comprehensive balance method" and " Comprehensive evaluation method "^29^.

### Comprehensive balance method

When adopting the comprehensive balance method for analysis, the following principles need to be followed: first, consider the main influencing factors of a certain goal and make them the first element to be considered; second, if the degree of influence of a factor on each goal is similar, select the level that appears more often according to the principle of majority rule; third, for relatively important optimization goals, priority should be given to meet their selection of factor levels; finally, for factors with insignificant influence, the cost should be considered when selecting their levels^30^.

The factor ranking and optimal combination under different objectives are organized in Table 15, where A is the number of discs, B is the disc diameter, C is the disc squeeze angle, D is the disc spacing, and the numbers represent their corresponding level numbers, same as below.

**Table 15:** Factor ranking and optimal combination.

Optimization objectives	Factor ranking	Optimal combination
I	BADC	A_3_B_3_C_1_D_3_
II	ABDC	A_3_B_3_C_1_D_3_
II	BACD	A_3_B_3_C_1_D_2_
IV	ADCB	A_3_B_2_C_1_D_3_
V	ACBD	A_3_B_1_C_1_D_1_
VI	BCAD	A_3_B_1_C_1_D_1_

There are six optimization objectives, compressive performance, compressive economic efficiency, uplift resistance, uplift economic efficiency, horizontal load bearing performance and maximum bending moment of the pile, which are abbreviated as I–VI.

According to the principles of the comprehensive balance method, the finalized optimal combination is A_3_B_3_C_1_D_3._

### Comprehensive evaluation method

In order to make a uniform evaluation of each objective, and because the scale of each objective to be examined is not consistent, a method is needed to convert a multi-objective problem into a single-objective problem. The comprehensive evaluation method can be used to quantify the different working conditions by scoring them and then implementing a qualitative ranking as a basis for comprehensive evaluation.

In order to make a uniform evaluation of each objective, and because the scale of each objective to be examined is not consistent, a method is needed to convert a multi-objective problem into a single-objective problem. The comprehensive evaluation method can be used to quantify the different conditions by scoring them and then implementing a qualitative ranking as a basis for comprehensive evaluation.

#### Queuing scoring method

The principle of the queuing scoring method is to rank and score each condition under the same optimization objective in order of performance, and finally its individual scores under different objectives are summed to get the total score^31^. Take DX4 as an example, it ranks eighth in compressive performance with a score of 2, seventh in compressive economic efficiency with a score of 3, ninth in uplift resistance with a score of 1, seventh in uplift economic efficiency with a score of 3, eighth in horizontal load bearing performance with a score of 2, and fourth in maximum bending moment with a score of 6. The six optimization objectives of DX4 score a total of 17, ranking eighth in the nine conditions. The six objectives of the nine conditions are counted according to the queuing scoring method and summarized in Table 16.

**Table 16:** Queuing scoring method score.

Pile type	Objective score						
	I	II	III	IV	V	VI	Total Score
DX1	1	1	2	1	7	9	21
DX2	4	2	3	2	1	4	16
DX3	6	6	6	4	3	2	27
DX4	2	3	1	3	2	6	17
DX5	5	4	4	5	4	5	27
DX6	9	8	7	7	5	3	39
DX7	3	5	5	8	8	8	37
DX8	7	9	8	9	9	7	49
DX9	8	7	9	6	6	1	37

The total score is used as the evaluation criterion for the range analysis, and Table 17 is obtained, which find that the order of influencing factors in descending order are: A, C, B, D; the optimal combination of factor parameters is A_3_B_3_C_1_D_3._

**Table 17:** Mean and range of queuing scoring method score.

	Factors			
	Number of discs	Disc diameter	Disc squeeze angle	Disc spacing
K1	21.333	25.000	36.333	28.333
K2	27.667	30.667	23.333	30.667
K3	41.000	34.333	30.333	31.000
R	19.667	9.333	13.000	2.667

#### Principal component analysis

Principal component analysis is a common technique for data downscaling and feature extraction. The basic idea is to transform the original variables into a new set of uncorrelated variables, which are called principal components. Through principal component analysis, the redundant information in the data is reduced and the main information of the data is retained. Principal component analysis is widely used in data mining, pattern recognition, signal processing and other fields^32^.

##### Data standardization

Before data standardization, the judgment of positive and negative indicators must be carried out, the larger the value of positive indicators the better, the smaller the value the worse, and the negative indicators are the opposite. Through the judgment, II and IV in this paper are positive indicators, and the remaining four indicators are negative indicators.

After importing the original data Table 18, due to the different magnitudes or magnitudes between the columns of data, the data need to be processed dimensionlessly, and the method of processing is the polar method with the following formula^33^:

12 $$ u_{ij} = \left\{ {\begin{array}{*{20}l} {\frac{{x_{ij} - \min \left( {x_{ij} } \right)}}{{\max \left( {x_{ij} } \right) - \min \left( {x_{ij} } \right)}}{,}\quad {\text{Positive indicators}}} \hfill \\ {\frac{{\max \left( {x_{ij} } \right) - x_{ij} }}{{\max \left( {x_{ij} } \right) - \min \left( {x_{ij} } \right)}}{,}\quad {\text{Negative indicators}}} \hfill \\ \end{array} } \right. $$

where $x_{ij}$ is the j-th indicator of the i-th condition; $u_{ij}$ is the result of dimensionless processing of $x_{ij}$; i = 1, 2,…, m; j = 1, 2,…, n; m and n are the number of working conditions and the number of indicators, m is taken as 9 and n is taken as 6 in this paper.

**Table 18:** Raw data.

Pile type	I	II	III	IV	V	VI
DX1	4.8973	1.8669	1.1499	1.1569	4.4409	84.57
DX2	2.6372	2.1839	0.9540	1.3153	10.0446	95.88
DX3	1.8271	2.3771	0.5459	1.3835	9.5718	96.11
DX4	3.5573	2.2433	1.7292	1.3483	9.8313	94.63
DX5	2.4388	2.3196	0.9034	1.4054	9.2417	95.73
DX6	1.1712	2.9279	0.3518	1.6420	8.8490	96.10
DX7	3.3416	2.3512	0.6937	1.6731	4.4001	86.42
DX8	1.2944	3.1645	0.3466	1.9194	4.2182	87.14
DX9	1.1726	2.5532	0.3458	1.4472	8.2052	97.20

The data obtained after standardization are shown in Table 19, and their sizes are controlled between [0, 1].

**Table 19:** Standardized data.

Pile type	I	II	III	IV	V	VI
DX1	0.0000	0.0000	0.0000	0.4187	0.9618	1.0000
DX2	0.2077	0.2443	0.6066	0.5604	0.0000	0.1045
DX3	0.2971	0.3932	0.8240	0.8554	0.0811	0.0863
DX4	0.2510	0.2901	0.3596	0.0000	0.0366	0.2035
DX5	0.3259	0.3489	0.6598	0.5969	0.1378	0.1164
DX6	0.6361	0.8177	1.0000	0.9957	0.2052	0.0871
DX7	0.6769	0.3732	0.4175	0.7485	0.9688	0.8535
DX8	1.0000	1.0000	0.9669	0.9995	1.0000	0.7965
DX9	0.3806	0.5289	0.9996	1.0000	0.3157	0.0000

##### Principal component analysis

Before using principal component analysis, it is necessary to first determine whether the data meet the requirements by subjecting the standardized data to KMO and Bartlett's sphericity tests. After calculation, the KMO is 0.594 and the p-value corresponding to the Bartlett's sphericity test is less than 0.05, indicating suitability for principal component analysis^34^.

The contribution of variance is obtained using principal component analysis in Table 20. A total of 2 principal components are extracted from the principal component analysis, and their corresponding eigenvalues are 3.249 and 2.236, with variance contribution rates of 54.153% and 37.26%, reaching a cumulative total of 91.413%.

**Table 20:** Contribution of variance.

PCA	Eigen values			Principal component extraction		
	Eigen	% of Variance	Cum. % of Variance	Eigen	% of Variance	Cum. % of Variance
1	3.249	54.153	54.153	3.249	54.153	54.153
2	2.236	37.260	91.413	2.236	37.260	91.413
3	0.431	7.181	98.594	–	–	–
4	0.056	0.938	99.531	–	–	–
5	0.020	0.336	99.867	–	–	–
6	0.008	0.133	100.000	–	–	–

The loading is an important indicator in the principal component analysis, which can be used to reflect the information extraction of each analysis term in the principal component. Table 21 shows the loadings of each analysis term in the principal components. According to communalities in Table 21, a strong correlation between the analyzed items and the principal components can be found, which means that the principal components can effectively extract the information in the studied items. An absolute value of the loadings greater than 0.4 indicates that the analysis term has a correspondence with the principal component.

**Table 21:** Loadings.

Items	Loadings		Communalities
	PCA1	PCA2	
I	0.904	−0.411	0.987
II	0.954	0.055	0.913
III	0.860	0.066	0.743
IV	0.861	0.368	0.876
V	0.114	0.980	0.973
VI	−0.168	0.982	0.992

##### Calculation of weights

The calculation of the weights is divided into the following three steps:

First, the linear combination coefficient matrix is calculated, as shown in Table 22. The linear combination coefficient is equal to the loadings divided by the square root of the corresponding eigen.

**Table 22:** Linear combination coefficient matrix.

Items	Component	
	Component1	Component2
I	0.502	−0.275
II	0.529	0.037
III	0.477	0.044
IV	0.478	0.246
V	0.063	0.655
VI	−0.093	0.657

Principal component score = linear combination coefficient matrix * standardized data. According to Table 20, Component1 score = 0.502*I + 0.529*II + 0.477*III + 0.478*IV + 0.063*V − 0.093*VI; Component2 score = − 0.275* I + 0.037*II + 0.044*III − 0.246*IV + 0.655*V + 0.657*VI.

From Table 20 and the component score formula, it can be seen that the first principal component, with a variance contribution of 54.15%, has moderate positive loadings in II and IV, and moderate negative loadings in I and II, so the first principal component can be called the vertical load bearing component of the squeezed branch pile; The second principal component, with a variance contribution of 37.26%, has moderate positive loadings on the V and VI, while the loadings on all other variables are small, so the second principal component can be called the horizontal load-bearing component of the squeezed branch pile. The first and second principal components together retain 91.41% of the information of the original index.

Second, the composite score coefficient is calculated by the formula: cumulative (linear combination coefficient * variance contribution rate)/cumulative variance contribution rate.

Third, the weights are calculated and all of them are normalized so that their weights sum to 1. This can be done by dividing the score for each objective by the sum of the six objective scores to obtain a weighting factor for each objective.

The results of the calculations are summarized in Table 23. The weights are ranked according to the magnitude of the calculated values, in the following order: I, IV, II, VI, V, III.

**Table 23:** Linear combination coefficient matrix and weights.

Name	Component1	Component2	Composite score coefficient	Weights
Eigen	3.249	2.236		
% of Variance	54.15%	37.26%		
I	0.5016	0.2752	0.1850	10.79%
II	0.5293	0.0368	0.3285	19.17%
III	0.4769	0.0443	0.3006	17.54%
IV	0.4776	0.2459	0.3831	22.35%
V	0.0631	0.6552	0.3044	17.76%
VI	0.0934	0.6567	0.2123	12.39%

##### Comprehensive evaluation

After obtaining the weights of each objective, the weights of each objective are multiplied by the standardized data in Table 19, and then expanded by one hundred times into a percentage scoring system, and the composite scores are obtained by arithmetic accumulation to obtain Table 24.

**Table 24:** Composite score by principal component analysis.

Pile type	I	II	III	IV	V	VI	Total score
DX1	0.0000	0.0000	0.0000	9.3588	17.0810	12.3900	38.8298
DX2	2.2410	4.6832	10.6391	12.5243	0.0000	1.2949	31.3824
DX3	3.2060	7.5374	14.4524	19.1172	1.4410	1.0693	46.8232
DX4	2.7081	5.5607	6.3075	0.0000	0.6499	2.5212	17.7474
DX5	3.5163	6.6879	11.5728	13.3413	2.4472	1.4421	39.0076
DX6	6.8638	15.6746	17.5400	22.2535	3.6442	1.0791	67.0552
DX7	7.3037	7.1548	7.3231	16.7297	17.2055	10.5752	66.2920
DX8	10.7900	19.1700	16.9597	22.3382	17.7600	9.8688	96.8868
DX9	4.1070	10.1390	17.5335	22.3500	5.6068	0.0000	59.7362

The mean and range analysis of the total score find that the order of influencing factors from largest to smallest is: A, C, B, D, and the optimal combination is A_3_B_3_C_1_D_2_.

#### Entropy weight method

The entropy weight method is a multi-indicator decision-making method designed to solve the problem of multiple indicators with different importance and interactions among them^35^. In information theory, the concept of "entropy" is used to consider the uncertainty of random variables, so the entropy weight method can be used to determine the weight of each indicator by calculating its contribution to the overall uncertainty^36^. If the information entropy of an objective is smaller, the more information that objective provides, the greater the weight will be.

The specific steps of the entropy weighting method are as follows^37^: first, the data matrix that has been normalized is denoted as $N = \left[ {x_{ij} } \right]_{m \times n}$, then the weight of the i-th sample value under the j-th objective to that objective is

13 $$ p_{ij} = \frac{{x_{ij} }}{{\sum\limits_{i = 1}^{m} {x_{ij} } }} $$

where i = 1, 2, …, $m$; j = 1, 2, …, $n$; $m$ and $n$ are the number of conditions and targets, respectively. $m$ is 9 and $n$ is 6 in this paper.

The entropy value for the j-th objective is calculated by the formula

14 $$ e_{j} = - k\sum\limits_{i = 1}^{m} {p_{ij} } \ln p_{ij} $$

where $k = 1/\ln m$. If $p_{ij} = 0$, it is replaced by 0.00001 for calculation, and $0 \le e_{j} \le 1$. The coefficient of variation of the j-th objective (column) is $0 \le e_{j} \le 1$, and the weight of the j-th objective (column) is

15 $$ \omega_{j} = \frac{{d_{j} }}{{\mathop \sum \limits_{j = 1}^{m} d_{j} }} $$

The calculation results for the six objectives are shown in Table 25.

**Table 25:** Entropy weighting method to calculate the weight results.

Items	Entropy value	Coefficient of variation	Weighting factor (%)	Weight Ranking
I	0.9248	0.0752	9.40	5
II	0.9009	0.0991	12.38	4
III	0.9326	0.0674	8.41	6
IV	0.8926	0.1074	13.41	3
V	0.7780	0.2220	27.73	2
VI	0.7704	0.2296	28.67	1

The degree of contribution of each objective to the whole using the geometric mean and linear weighting method is given by

16 $$ U = \sum\limits_{j = 1}^{n} {w_{j} } u_{ij} ,\sum\limits_{j = 1}^{n} {w_{j} } = 1 $$

where $U$ is the combined score value of the six objectives, the total score in Table 26 is expanded by a factor of one hundred.

**Table 26:** Entropy method composite score.

Pile type	Total scores
DX1	60.9550
DX2	20.5888
DX3	30.7847
DX4	15.8233
DX5	28.0939
DX6	46.0512
DX7	75.8672
DX8	93.8808
DX9	40.6968

The mean and range analysis is performed on the total scores in Table 26 to obtain Table 27.

**Table 27:** Mean and range of entropy weight method score.

	Factors			
	Number of discs	Disc diameter	Disc squeeze angle	Disc spacing
K1	37.443	50.882	66.962	43.249
K2	29.989	47.521	25.703	47.502
K3	70.148	39.178	44.915	46.830
R	40.159	11.704	41.259	4.253

According to the orthogonal test to analyze the total score of each working condition, it is found that the order of influencing factors from the largest to the smallest is C, A, B, D, and the optimal combination of factor parameters is A_3_B_3_C_1_D_2._

#### Analytic hierarchy process

The Analytic Hierarchy Process is a quantitative approach to decision analysis that was proposed by American mathematician Thomas L. Saaty in the 1970s^38^. This method can decompose a complex decision-making problem into several hierarchical structures with inherent logical relationships layer by layer, and then determine the importance weights of each hierarchical factor by comparing them two by two, and finally arrive at a comprehensive evaluation result.

The specific steps of the analytic hierarchy process are as follows^39^:

First, a judgment matrix is constructed, and a comparison between two method is used to construct a judgment matrix for the factors in each level. Make judgment matrix Table 28 based on the working environment and empirical judgment of the squeeze branch pile.

**Table 28:** Judgment matrix.

	I	II	III	IV	V	VI
I	1	3	6/5	6	3/2	2
II	1/3	1	2/5	2	1/2	2/3
III	5/6	5/2	1	5	5/4	5/3
IV	1/6	1/2	1/5	1	1/4	1/3
V	2/3	2	4/5	4	1	4/3
VI	1/2	3/2	3/5	3	3/4	1

The second step is to calculate the eigenvector, the eigenvalue and the weight calculation. The calculation is performed using the eigenvalue method for the judgment matrix, and the results of analytic hierarchy process are obtained in Table 29.

**Table 29:** Results of Analytic Hierarchy Process.

Items	Eigenvector	Weight (%)	Maximum Eigenvalue	CI
I	1.714	28.571	6.000	0.000
II	0.571	9.524		
III	1.429	23.810		
IV	0.286	4.762		
V	1.143	19.048		
VI	0.857	14.286		

Finally, a consistency test is performed. In the process of calculating the weight vector, the consistency of the judgment matrix needs to be checked. If the judgment matrix has a large misalignment, then the calculated weight vector may produce unreasonable results. Therefore, the consistency ratio needs to be used to assess the consistency of the judgment matrix, and if the consistency ratio is less than 0.1, the judgment matrix is considered to have good consistency. Where the general consistency index $CI = \left( {\lambda_{\max } - n} \right)/(n - 1)$ for the judgment matrix, and the maximum eigenvalue $\lambda_{\max }$ in this paper is equal to the matrix order n, so $CI = 0$.

$CR = CI/RI$, for a 6th order judgment matrix, $RI = 1.26$. By calculating $CR = 0 < 0.1$, so the judgment matrix satisfies the consistency test.

Through the above steps, the weights of each factor can be obtained, and thus a comprehensive score can be given to each pile. The scores are shown in Table 30.

**Table 30:** Analytic Hierarchy Process composite score.

Pile type	Total scores
DX1	34.6048
DX2	26.8625
DX3	38.7010
DX4	22.0993
DX5	35.4710
DX6	59.6610
DX7	67.0481
DX8	96.3020
DX9	50.4848

The mean and range analysis is performed on the total scores of Table 30 to obtain Table 31.

**Table 31:** Mean and range of analytic hierarchy process score.

	Factors			
	Number of discs	Disc diameter	Disc squeeze angle	Disc spacing
K1	33.389	41.251	65.523	40.187
K2	39.077	52.879	33.149	51.191
K3	71.278	49.616	47.073	52.367
R	37.889	11.628	30.374	12.180

The mean and range analysis of the total score find that the order of influencing factors from largest to smallest is: A, C, D, B, and the optimal combination is A_3_B_2_C_1_D_3_.

### Aggregate judgment

The results obtained from the various methods mentioned above are summarized to obtain Table 32.

**Table 32:** Factor ranking and optimal combination of different methods.

Analysis method	Factor ranking	Optimal combination
Comprehensive balance method	–	A_3_B_3_C_1_D_3_
Queuing scoring method	ACBD	A_3_B_3_C_1_D_3_
Principal component analysis	ACBD	A_3_B_3_C_1_D_2_
Entropy weight method	CABD	A_3_B_3_C_1_D_2_
Analytic hierarchy process	ACDB	A_3_B_2_C_1_D_3_

Considering five different methods to obtain the factor ranking and optimal combination, A_3_B_3_C_1_D_3_is judged to be the optimal combination, i.e., the number of support discs is 4, the disc diameter is 2.5D, the disc squeeze angle is 35°, and the disc spacing is 3D.

The conclusions obtained from the various methods are basically the same, indicating that the selection of factors and levels is basically the same for the various methods as long as the principles of evaluating the test indicators remain the same.

## Conclusion

The squeezed branch pile has the advantages of easy construction, low cost and wide applicability of piling process, which is widely used in the field of pile foundation engineering. The significance of studying the squeezed branch piles is to further grasp their working mechanism, optimize the design parameters, improve the construction quality and efficiency, and explore their applicability in different engineering environments to meet the demand for foundation strengthening and improvement in construction projects.

The findings and main conclusions of this paper are as follows:

(1) The factors and levels of geometric parameters of the squeezed branch piles are determined by linking with the actual engineering practice, and nine different parameters of the squeezed branch piles are obtained using orthogonal test design.

(2) The order of influence of various factors on the vertical settlement of the squeezed branch pile from the largest to the smallest is: disc diameter, number of discs, disc spacing, and disc squeeze angle. The order of influence of the four factors on the economic efficiency of the squeezed branch pile is from the largest to the smallest: number of discs, disc diameter, disc spacing, disc squeeze angle. The optimal combination of 4 discs, 2.5D disc diameters, 35° disc squeeze angle, and 3D disc spacing, DX10 has the optimal combination of both compressive resistance capabilities.

During the whole loading stage, the load shared by the discs has been 43–55%. With the increase of depth, the pile body axial force gradually decreases, and this reduced axial force is borne by the discs and transferred to the soil below the discs. As the load increases, the reduction of the pile axial force at the discs becomes larger. By analyzing the cloud diagram, it is found that the soil near the discs moves much more compared with the soil in other parts, which indicates that the soil on the lower side of the discs is sufficiently compressed by the load transferred from the discs, and the soil on the lower side of the pile end and the soil on the lower side of the discs are the main areas of compressive stress concentration.

(3) The influence of various factors on the vertical uplift of the squeezed branch pile in the order of the four factors: disc diameter, number of discs, disc squeeze angle, disc spacing. The optimal combination of factor parameters is 2.5D disc diameters, 4 discs, 35° disc squeeze angle, 2.5D disc spacing. The uplift loads borne by the disc end resistance account for 58.6% to 79.3% of the total, where the uppermost disc provides the highest bearing capacity in all bearing stages, while the pile side frictional resistance bears less, accounting for 20.7% to 41.3% of the total.

The order of influencing factors for the horizontal displacement of the squeezed branch pile is from the largest to the smallest: number of discs, disc squeeze angle, disc diameter, disc spacing; and the order of influencing factors for the maximum bending moment of the squeezed branch pile is from the largest to the smallest: disc diameter, disc squeeze angle, number of discs, disc spacing. Two sets of optimization objectives are simultaneously optimized for the number of 4 discs, disc diameter of 1.5D, disc squeezing angle of 35°, and disc spacing of 2D. The horizontal displacement of the pile decreases with the increase of the pile depth, the horizontal displacement of the ground soil in front of the pile decreases with the increase of the radial distance from the pile, and the uplift of the ground soil in front of the pile increases first and then decreases with the increase of the radial distance. The maximum stress of the soil around the pile appears on the left side of the pile end and the right side of the middle end of the pile. The displacement variation of the soil around the pile is concentrated on the right side of the pile top near the ground, and a small part appears on the left side of the pile end.

(4) The six optimization objectives of compressive performance, compressive economic efficiency, uplift performance, uplift economic efficiency, maximum horizontal displacement and maximum bending moment of the pile are analyzed using multi-objective optimization design, with specific methods such as comprehensive balance method, queuing scoring method, principal component analysis, entropy weight method and analytic hierarchy process. By judgment, the squeezed branch pile with 4 discs, 2.5D disc diameter, 35°disc squeeze angle and 3D disc spacing is considered to be the optimal combination considering all optimization objectives.

## Data Availability

The data supporting this study's findings are available from the corresponding author upon reasonable request.
